# Comparative transcriptomics reveals *CrebA* as a novel regulator of infection tolerance in *D*. *melanogaster*

**DOI:** 10.1371/journal.ppat.1006847

**Published:** 2018-02-02

**Authors:** Katia Troha, Joo Hyun Im, Jonathan Revah, Brian P. Lazzaro, Nicolas Buchon

**Affiliations:** Cornell Institute of Host-Microbe Interactions and Disease, Department of Entomology, Cornell University, Ithaca, New York, United States of America; Stanford University, UNITED STATES

## Abstract

Host responses to infection encompass many processes in addition to activation of the immune system, including metabolic adaptations, stress responses, tissue repair, and other reactions. The response to bacterial infection in *Drosophila melanogaster* has been classically described in studies that focused on the immune response elicited by a small set of largely avirulent microbes. Thus, we have surprisingly limited knowledge of responses to infection that are outside the canonical immune response, of how the response to pathogenic infection differs from that to avirulent bacteria, or even of how generic the response to various microbes is and what regulates that core response. In this study, we addressed these questions by profiling the *D*. *melanogaster* transcriptomic response to 10 bacteria that span the spectrum of virulence. We found that each bacterium triggers a unique transcriptional response, with distinct genes making up to one third of the response elicited by highly virulent bacteria. We also identified a core set of 252 genes that are differentially expressed in response to the majority of bacteria tested. Among these, we determined that the transcription factor *CrebA* is a novel regulator of infection tolerance. Knock-down of *CrebA* significantly increased mortality from microbial infection without any concomitant change in bacterial number. Upon infection, *CrebA* is upregulated by both the Toll and Imd pathways in the fat body, where it is required to induce the expression of secretory pathway genes. Loss of *CrebA* during infection triggered endoplasmic reticulum (ER) stress and activated the unfolded protein response (UPR), which contributed to infection-induced mortality. Altogether, our study reveals essential features of the response to bacterial infection and elucidates the function of a novel regulator of infection tolerance.

## Author summary

How does an organism survive infection? How generic or specific is the host response to diverse pathogens? To address these questions, we infected fruit flies with 10 different bacteria that vary in their ability to kill flies and measured changes in global gene expression. In general, we found that the host response is highly specific to individual bacteria. However, we also discovered a set of genes that changed expression in response to the majority of bacteria tested. Among these genes, we determined that the transcription factor *CrebA* is a novel regulator of the host response to infection. We found that upon infection, the immune system induces the expression of *CrebA*. *CrebA*-deficient flies are more likely to die from infection despite carrying the same number of bacteria as wildtype flies. *CrebA* is expressed in the fat body, an organ analogous to the mammalian liver and adipose tissues, where it regulates the transcription of multiple secretory pathway genes. Loss of *CrebA* during infection triggers endoplasmic reticulum (ER) stress (a type of cellular stress), which is sufficient to sensitize flies to infection. These results suggest that the immune system can modulate host physiology to prevent the deleterious effect of infection-associated cellular stress.

## Introduction

To combat infection, a host activates a combination of immune and physiological responses. While detection of microbial presence is sufficient to stimulate the innate immune response, physiological responses to infection occur as a consequence of microbial growth and virulence, and can therefore be very specific to the particular bacterium the host interacts with. Despite a growing body of literature on immunity, our knowledge of the different host processes that are activated or repressed in response to infection, and of how such responses contribute to host survival, remains limited. To identify new biological processes required to survive infection and to determine how specific or generic the immune and physiological responses to infection are, we surveyed changes in the transcriptome of *Drosophila melanogaster* in response to infection with 10 bacteria that span the spectrum of virulence.

*Drosophila* is a leading model system for studying how hosts respond to infection at the organismal level. To overcome infection, the fly relies on cellular and humoral innate immune responses. The cellular response consists of phagocytosis and encapsulation [1,2]. The humoral response includes the pro-phenoloxidase cascade, which leads to the generation of reactive oxygen species and clotting, as well as the production of antimicrobial peptides (AMPs) primarily by the fat body, an organ functionally analogous to the liver and adipose tissues of mammals [3–5]. In the early 2000s, microarray studies characterizing the transcriptional response to bacterial infection were conducted in *Drosophila* [6–8]. These experiments were based on infection with two non-pathogenic bacteria, *Micrococcus luteus* and *Escherichia coli*. This approach successfully identified a set of genes that are differentially expressed upon infection, which became known as the *Drosophila* Immune-Regulated Genes (DIRGs). A majority of the DIRGs were functionally assigned to specific aspects of the immune response—phagocytosis, antimicrobial peptide synthesis, and production of reactive oxygen species among others [6]. These studies also confirmed that the Toll and Imd pathways are the major regulators of the immune response in *Drosophila*, and that both pathways direct expression of the majority of DIRGs [7]. In this model, the host response depends on the sensing of two microbe-associated molecular patterns (MAMPs): Lys-type peptidoglycan from Gram-positive bacteria, which activates the Toll pathway, and DAP-type peptidoglycan from Gram-negative bacteria, which induces the Imd pathway [9–11]. Upon activation, each pathway goes on to regulate a subset of DIRGs.

More recently, new findings have expanded our insight into the *Drosophila* response to infection. First, the Toll and Imd pathways can also be activated by virulence factors and damage-associated molecular patterns (DAMPs) [12–16]. Additionally, biological processes that would not be considered as classic immunological responses, such as tissue repair and regulation of metabolism, are clearly modulated by pathogenic infection [17–20]. These observations beget the idea that microbial virulence—the relative capacity of a microbe to cause damage in a host—could be an important factor in shaping the host response, and suggest that survival from pathogenic infections may require additional biological processes beyond those that are currently known [21].

In this study, we aimed to identify a comprehensive list of genes regulated by pathogenic and avirulent infections, and to determine what responses are general or specific to each infection. To that purpose, we used RNA-seq to profile the *D*. *melanogaster* transcriptomic response to systemic infection with 10 different species of bacteria that vary in their ability to grow within and kill the host. We found that each bacterium elicits a unique host transcriptional response. However, we also identified a small set of core genes that were differentially regulated by infection with the majority of microbes. These genes are involved in a variety of immune and non-immune functions, and a fraction of them remained highly expressed even after bacteria were cleared from the host. Among the core genes was *CrebA*, a Creb3-like transcription factor. *CrebA* expression is upregulated through both Toll and Imd signaling in the fat body following infection. Knockdown of *CrebA* significantly increased mortality from bacterial challenge but did not alter bacterial load, indicating that *CrebA* contributes to host tolerance of infection. *CrebA* regulates multiple genes involved in the secretory pathway, and the loss of *CrebA* triggered ER stress upon infection. This suggests that the *CrebA* tolerance phenotype may arise through protection from cellular stress during the rapid and dramatic response to infection.

## Results

### Identification of bacteria with different virulence levels and peptidoglycan types

**Fig 1:**
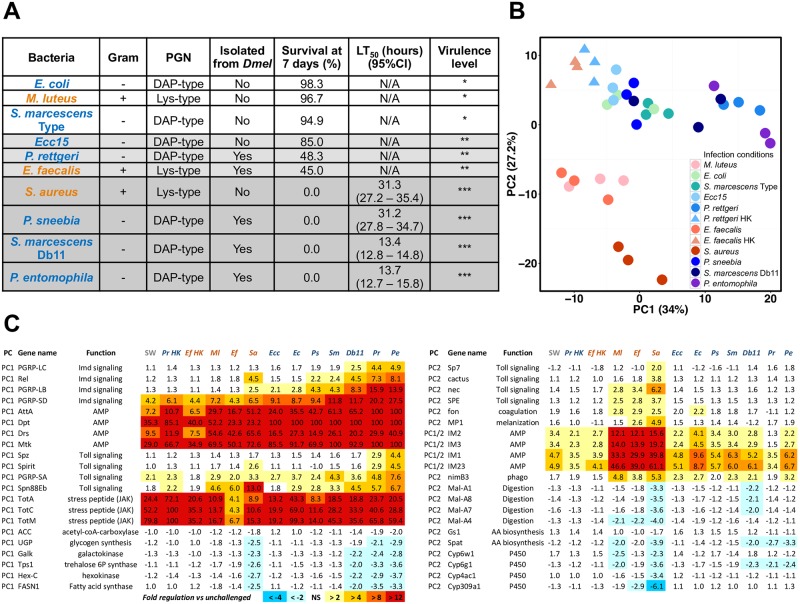
Major parameters influencing the global response to infection. (A) List of bacteria used in the RNA-seq experiment, including Gram classification, type of bacterial peptidoglycan (PGN), source of isolate, percent survival at 7 days post-infection, median lethal time (LT_50_) for each bacterium, and assignment into broad virulence categories. (B) PCA plot showing the first two principal components of the 12 h dataset. Red and orange (warm) colors indicate infections with Lys-type PGN bacteria, while green, blue and purple (cool) colors indicate infections with DAP-type PGN bacteria. Circles indicate infection with live bacteria and triangles denote inoculation with heat-killed bacteria. (C) Genes that contribute the most to PC1 (left column), PC2 (right column), or both PCs (right column) are presented with their associated level of expression change (fold change) at 12 h post-infection. Warm colors indicate the degree of transcriptional induction, while cool colors show the extent of transcriptional downregulation.

We began by assembling a panel of bacteria to probe the host response to infection. We selected bacteria that span the spectrum of virulence (from 0% to 100% mortality), focusing on microbes that are commonly used by the *D*. *melanogaster* research community and ensuring that we included bacteria with Lys-type or DAP-type peptidoglycan (PGN) in each virulence level. To assess the relative virulence of each bacterium, we measured host survival and bacterial load over time following infection (Fig 1A, S1 and S2 Figs). The bacteria with the lowest levels of virulence—*Escherichia coli* (*Ec*), *Micrococcus luteus* (*Ml*), and the Type strain of *Serratia marcescens* (*Sm*)—caused less than 10% mortality and did not grow past initial inoculum levels in the host. Bacteria exhibiting intermediate levels of virulence—*Pectinobacterium* (previously known as *Erwinia*) *carotovora 15* (*Ecc15*), *Providencia rettgeri* (*Pr*), and *Enterococcus faecalis* (*Ef*)—showed the ability to proliferate within the host and killed 15% to 55% of infected hosts. Highly virulent bacteria—*Staphylococcus aureus* (*Sa*), *Providencia sneebia* (*Ps*), *Serratia marcescens* strain Db11 (Db11), and *Pseudomonas entomophila* (*Pe*)—caused 100% mortality in less than 96 h (Fig 1A). *M*. *luteus*, *E*. *faecalis*, and *S*. *aureus* are Gram-positive bacteria (Lys-type PGN); all others are Gram-negative (DAP-type PGN).

Bacterial load time course experiments revealed differences between bacterial species in their ability to grow and persist within the host. For example, only *M*. *luteus* and *Ecc15* were eliminated from the host (i.e. their levels fall below our detection threshold of ~30 CFU/fly) by 132 h post-infection. In the case of *Ecc15*, most but not all hosts were able to clear the infection (S2A and S2F Fig). Neither *E*. *coli* nor *S*. *marcescens* Type increased in density, but the bacteria persisted inside the host at ~2^10^ bacteria/fly even after 5 days of infection (S2D and S2E Fig). *P*. *rettgeri* and *E*. *faecalis* grew during the first 24 h of infection, killing a fraction of the hosts. The flies that survived these infections remained chronically infected with ~2^10^ to 2^13^ bacteria per fly (S2B and S2G Fig) for at least 5.5 days. *P*. *entomophila*, *S*. *aureus*, *S*. *marcescens* Db11, and *P*. *sneebia* all grew monotonically in the host until death occurred (S2C, S2H, S2I and S2J Fig), causing complete mortality within 96 h (S1E, S1J, S1K and S1L Fig).

Having assembled our panel of bacteria, our next goal was to select relevant time points for transcriptomic analysis. Using our survival and bacterial load data, we identified three time points that are characteristic of different stages of infection: 12, 36, and 132 h. At 12 h post-infection, all flies remain alive, and they face the initial growth of microbes. Thirty-six hours represents an intermediate time point during infection, after the highly virulent bacteria have killed most or all flies and the moderately virulent bacteria have killed 15% to 55% of infected hosts. Finally, at 132 h post-infection (5.5 days), surviving flies are chronically infected with moderate to low levels of bacteria.

### A diverse, partly specific *Drosophila* response to infection

To identify novel biological processes required to survive systemic infection, and to assess the level of specificity of the *Drosophila* response to microbes, we used RNA-seq to profile the *D*. *melanogaster* transcriptome after infection with each of our 10 experimental bacteria. We additionally included the following controls: unchallenged flies (UC), flies challenged with a sterile wound (SW), and flies inoculated with heat-killed *E*. *faecalis* (*Ef* HK) or heat-killed *P*. *rettgeri* (*Pr* HK). The purpose of the controls was to distinguish the response to live bacteria from that to aseptic injury and/or inert bacterial compounds (MAMPs) provided by the injection of dead bacteria. The expression value dataset for the entire experiment can be downloaded or accessed online in our associated database Flysick-seq (http://flysick.buchonlab.com)

**Fig 2:**
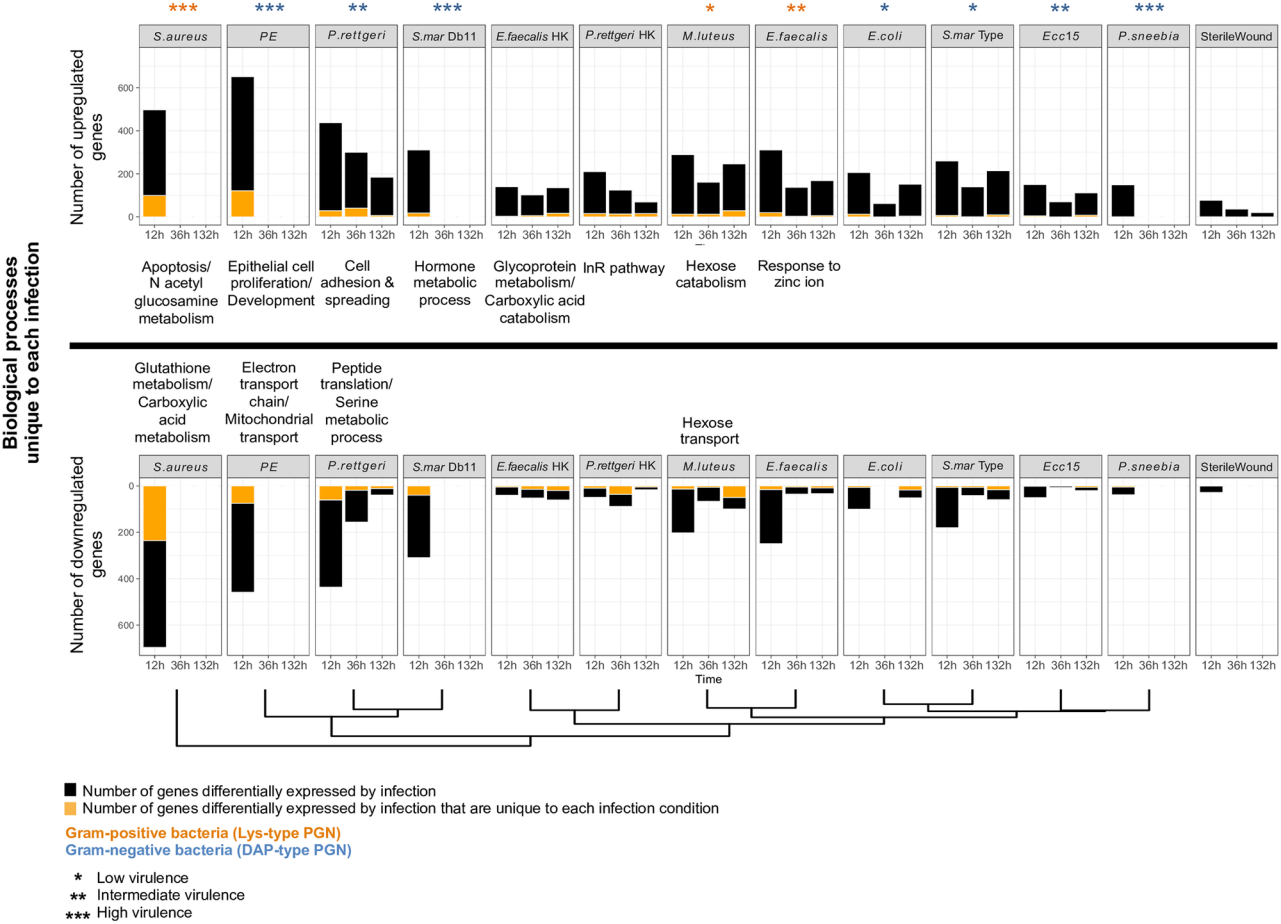
The host response to bacterial infection is diverse. (A) Number of genes differentially regulated by each infection (black bars) and number of genes differentially expressed by infection that are unique to each individual bacterium (orange bars). This information is listed separately by time point for each infection. Upregulated and downregulated genes are above and below the horizontal demarcation line, respectively. The biological processes regulated exclusively in response to each individual bacterium, if any, are listed adjacent to each bacterium. These biological processes are also separated by the horizontal demarcation line depending on whether they are upregulated (above) or downregulated (below) by each infection. The relative level of virulence of each bacterium is indicated by the number of stars: *Low virulence, **Intermediate virulence, ***High virulence. The type of bacterial peptidoglycan is indicated by the color of the stars: orange (bacteria with Lys-type PGN) and blue (bacteria with DAP-type PGN). The clustering of infection conditions (shown at the bottom of the graph) is based on the similarities of the expression patterns measured at 12 h.

We first determined the overall transcriptomic differences between flies infected by each of the 10 bacteria. Principal component analysis (PCA) showed that all three biological replicates clustered together, indicating good replicability of the response for each pathogen (illustrated in Fig 1B and 1C for the 12 h time point and S3A Fig for the full data set). In total, we identified 2,423 genes (13.7% of the genome) that were differentially expressed upon infection. Of these, 1,286 genes were upregulated and 1,290 genes were downregulated in response to at least one bacterial infection and time point (Fig 2A and S3B Fig). Out of the total number of genes differentially regulated by all 10 live infections, more genes were upregulated than downregulated; 6.1% of the 1,286 upregulated genes were induced in all bacterial infections, while only 0.6% of the 1,290 downregulated genes were repressed by all 10 bacteria (S3B Fig). We also determined that 51.1% of the downregulated genes were repressed in only one bacterial infection, while 38.6% of the upregulated genes were induced by a single bacterial condition (S3B Fig). These data suggest that the host response to infection is highly specific to individual bacteria, but that there is also a core set of genes that are differentially expressed during most bacterial infections. Additionally, our data showed that downregulated genes tend to be unique to each infecting bacterium, perhaps reflecting the singular consequences of each infection to host physiology (S3B Fig).

In general, the largest number of differentially expressed genes was observed at 12 h post-infection. However, a substantial number of genes continued to be differentially regulated at 36 h and 132 h post-inoculation (Fig 2A), presumably in part because the hosts continue to carry their bacterial infections at these later time points and/or because infection induces long-term changes in host physiology. Samples for the 36 h and 132 h time points were not available for infections with the highly virulent bacteria because they rapidly killed all their hosts. For the remaining infections, however, the number of upregulated genes at 12 h after infection was 1.6 times higher than the average number of genes that continued to be induced at 36 h and 132 h post-infection. Likewise, there were 2.8 times as many downregulated genes at 12 h post-infection than there were at later time points. These results demonstrate that the early transcriptional response to infection is larger than the sustained one, probably because the early response includes both an injury-induced transcriptional regulation and an aggressive initial immune response that is not yet tuned to bacterial titer or growth state within the host [22].

### Major axes of variation in the transcriptional response to infection

We sought to investigate the source of differences in the host response to various infections. We began by looking at the number of genes regulated by the host in response to each bacterium. The number of differentially regulated genes fluctuated considerably across bacterial infections (Fig 2A). Flies inoculated with heat-killed *E*. *faecalis* and *P*. *rettgeri*, as well as flies challenged with avirulent bacteria, such as *E*. *coli* and *M*. *luteus*, induced the lowest number of genes. However, the number of genes regulated in the host did not directly correlate with the level of bacterial virulence. For example, despite the fact that both bacteria rapidly killed all flies, infection with *S*. *aureus* differentially regulated the expression of 1,193 genes, while *P*. *sneebia* infection altered the transcription of only 187 genes (Fig 2A). In addition, there was a large variability in the number of genes regulated in response to different benign bacteria. Across all time points, *M*. *luteus* infection changed the expression of 794 genes, while *E*. *coli* infection affected only 446 genes (Fig 2A). These results indicate that the breadth and the specificity of the host transcriptomic response is largely independent of virulence.

Next, we aimed to identify specific genes that underlie the transcriptomic differences in response to distinct infections. We focused on the first two principal components of our PCA analysis (Fig 1B), which respectively explain 34.0% and 27.2% of the variance in gene expression. We found that 73 of the top 100 genes contributing to the first principal component (PC1) and 75 of the 100 genes contributing most to the second principal component (PC2) are known targets of the Toll or Imd pathways (Fig 1C and S4 Fig), confirming that these two pathways are key regulators of the specificity of the host response [7]. The genes that contributed most to PC1 included antimicrobial peptide genes (*Dpt*, *AttA*, *Drs*, and *Mtk*) as well as signaling components of the Toll (*Spz* and *PGRP-SA*) and Imd (*PGRP-LC*, *PGRP-SD*, *PGRP-LB*, and *Rel*) pathways themselves (Fig 1C). Additionally, the expression of Turandot genes, stress peptides regulated by the JAK-STAT pathway, was strongly variable between infections, indicating that differential activation of the JAK/STAT pathway also contributes to PC1. Interestingly, metabolic genes involved in lipid synthesis (*ACC*), the Leloir pathway (*Galk*), and trehalose and glycogen synthesis (*Tps1*, *UGP*, and *Hex-C*) were downregulated to different levels depending on the infection, indicating that different bacteria alter host metabolism in unique ways. In general, PC1 appeared to reflect the transcriptional magnitude of the response to infection. Genes that contributed most to PC2 include target genes of the Toll pathway, including melanization and coagulation-related genes (*MP1* and *fondue*) (Fig 1C), as well as immune-induced proteins of the IM cluster. PC2 also included genes downregulated by infection that are involved in sugar digestion (i.e. the *Maltase* cluster), as well as P450 enzymes known for their functions in oxidoreduction reactions (i.e. *Cyp* genes). Flies infected with Gram-positive bacteria (Lys-type PGN) and Gram-negative bacteria (DAP-type PGN) were separated from each other on PC2, confirming that the type of bacterial peptidoglycan is a major parameter influencing the global response to infection (Fig 1B, S3A Fig) [7]. A heatmap showing the expression level of genes that contribute the most to each PC can be found in S4 Fig.

Subsequently, we asked whether any differentially regulated genes were unique to a specific bacterial condition. We defined unique genes as those that significantly changed their expression in one and only one infection condition, regardless of time points, thus reflecting the response to a particular bacterium rather than temporal variations in the response to this bacterium. Without exception, we found that infection with each bacterium regulates an exclusive set of genes. The number of uniquely regulated genes varied dramatically across bacterial infections (Fig 2A). For instance, *P*. *sneebia* infection resulted in unique regulation of only 6 genes, whereas *S*. *aureus* infection exclusively regulated 336 genes. In order to determine what portion of the host response is specific to individual bacteria, we calculated the percentage of differentially expressed genes that were unique to each infection (S3C Fig). We found that this number also differs widely between bacteria. For instance, 20.1% of genes upregulated in response to *S*. *aureus* were exclusive to this infection, while only 7.1% of genes upregulated by *E*. *faecalis* infection were unique to this condition. Evaluating Gene Ontology (GO) terms associated with the genes uniquely altered by individual infections revealed bacteria-specific responses in some infection conditions (Fig 2A). For example, *S*. *aureus* infection induced apoptosis-related genes and downregulated genes involved in glutathione and carboxylic acid metabolism. In contrast, infection with *P*. *entomophila* upregulated genes involved in epithelial cell proliferation and strongly decreased the expression of genes associated with cellular respiration and the electron transport chain. At the same time, infection by *P*. *rettgeri* specifically downregulated genes involved in the translation machinery (Fig 2A). All the GO gene categories we identified are linked to stress responses that aim to maintain cell homeostasis (cell death and tissue repair) or metabolic homeostasis, suggesting that the unique physiological and virulence interactions of each bacterium with the host induce a specific set of organismal responses. Altogether, our results demonstrate that the host response to infection is shaped by a combination of immune potency, metabolic impact, and physiological alteration.

### Identification of a core host response to infection

**Fig 3:**
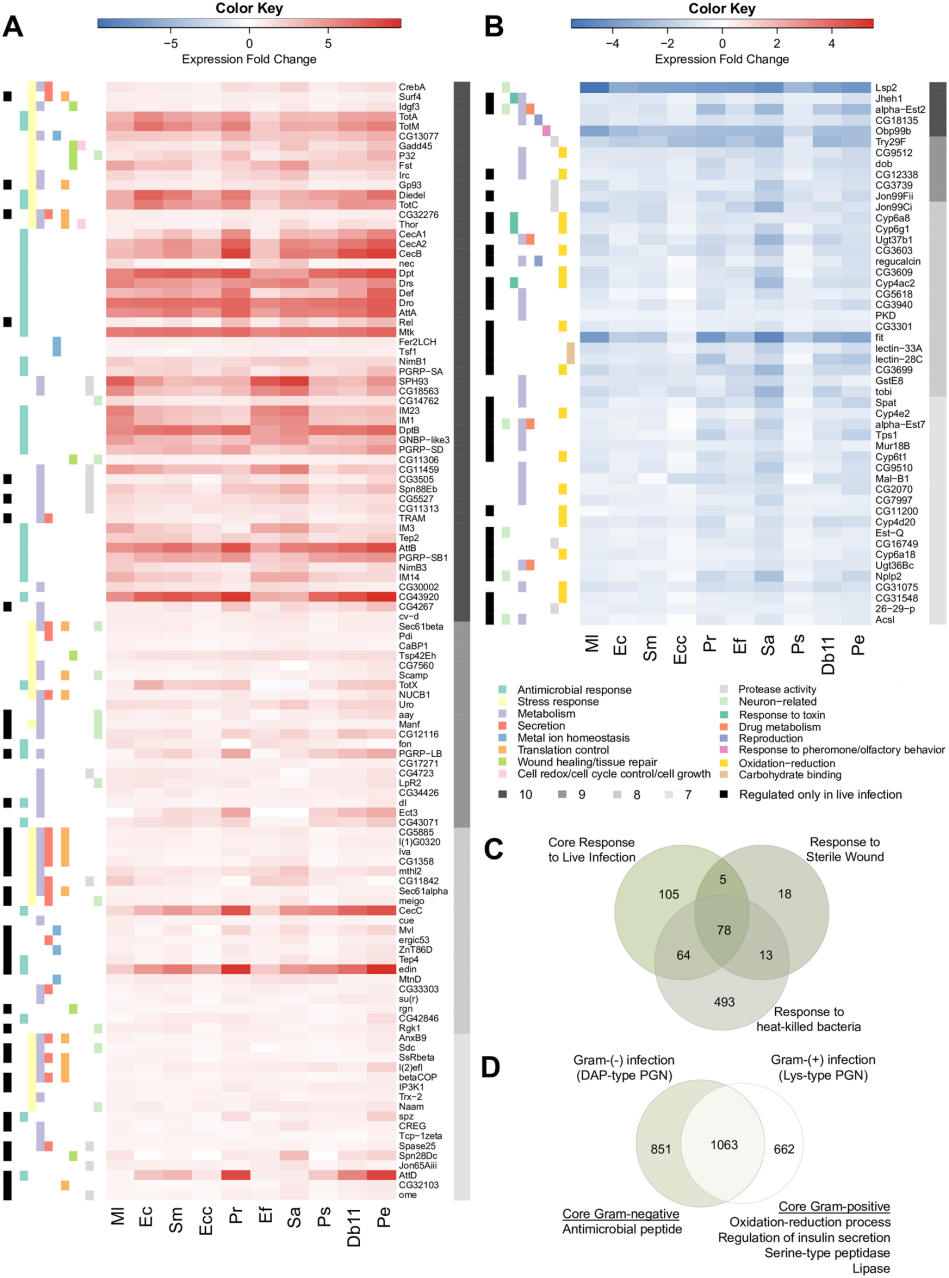
Systemic infection triggers a core host response. Heatmap showing the expression level (log_2_ fold change) of selected core upregulated (A) and downregulated (B) genes for all 10 bacteria. Core genes are differentially expressed in response to infection by 7 or more bacteria. A gray scale on the right side of each heatmap indicates the number of bacterial infections that significantly change the expression of a given gene (dark gray = 10, medium gray = 9, light gray = 8, and very light gray = 7). A color scale on the left side of each heatmap denotes the functional categories that each gene belongs to, and the legend for each color is listed at the bottom of the graph. (C) Venn diagram showing the intersection of core genes, sterile wound genes, and genes differentially regulated by challenge with heat-killed bacteria. (D) Venn diagram indicating the number of genes differentially regulated only in response to infection with Lys-type peptidoglycan (PGN) bacteria (right), DAP-type PGN bacteria (left), and those genes differentially expressed by challenge with both types of bacteria. GO terms associated with genes exclusively regulated by infection with Lys-type PGN bacteria or DAP-type PGN bacteria are listed.

Next, we set out to identify the core set of genes that are regulated in response to most or all bacterial infections. We defined the core genes as those that are differentially expressed in response to 7 or more bacteria on at least one time point post-infection. We set the cutoff at 7 bacteria because we were concerned that requiring differential expression in response to all 10 infections would be overly restrictive. Specifically, we had reservations about the artificial omission of genes in cases where the bacteria are rapidly cleared from all or most hosts (e.g. *M*. *luteus* and *Ecc15*) and in cases where the bacterium might suppress or evade the canonical response (e.g. *P*. *sneebia*; [23]). Using these criteria, we identified a core response of 252 genes. This included 166 upregulated genes (Fig 3A and S1 Table) and 86 downregulated genes (Fig 3B and S1 Table). The set of core genes is fairly robust to the criteria for inclusion, decreasing only to 135 genes induced and 54 genes repressed when inclusion required differential expression in response to 8 of the bacterial conditions. Similarly, the numbers increased only to 216 genes induced and 136 repressed when inclusion was relaxed to 6 of the bacterial infections.

Within the core, 78 genes were also regulated in response to sterile wound alone or to challenge with heat-killed bacteria (Fig 3C). Most of the genes regulated by injury were also regulated by challenge with live or dead bacteria (96/114 genes), which is congruent with the fact that the infection method inherently inflicts injury. However, the core response to live infection was markedly distinct from the response to heat-killed bacteria. Of our core genes, ~40% (105/252 genes) were differentially expressed in response to live infections but not in response to challenge with heat-killed bacteria. Moreover, we found 493 genes that were differentially regulated by treatment with heat-killed bacteria but were not part of the core response to live infection (Fig 3C). Of those 493 genes, 164 were uniquely regulated in response to heat-killed bacteria and not in response to any live infection (S5A Fig). To determine whether genes exclusively regulated in response to heat-killed bacteria are simply artifacts of weak statistical detection, we relaxed the cutoff to a False Discovery Rate (FDR) <0.1 for classifying a gene as differentially expressed during infection. Even with this more lenient threshold, 61.6% of the 164 genes that were uniquely regulated in response to heat-killed bacteria were still not differentially regulated in response to any live infection. Our results, therefore, not only show that the response to live infections is fundamentally different from the biological challenges that simple injury and immune activation pose, but also demonstrate that challenge with dead bacteria induces a response that does not occur as a consequence of infection by live bacteria.

In 2001, a study identified a set of genes that are differentially expressed after infection with a combination of *E*. *coli* and *M*. *luteus* [6]. These genes became known as *Drosophila* Immune-Regulated Genes (DIRGs). We compared our set of 252 core response genes to the 381 DIRGs and found that only 84 of them were previously identified as DIRGs (S5B Fig). Intriguingly, the DIRGs identified in the previous study included 279 genes that were neither in our core response nor regulated by challenge with heat-killed bacteria (S5B Fig), and 246 of these DIRGs were not induced in the present study even by infection with *M*. *luteus* or *E*. *coli* (S5C Fig). These discrepancies may originate from differences in *Drosophila* genotype or rearing conditions, bacterial genotype, or experimental variation. Alternatively, they could imply that infection with a mixture of two bacteria can lead to the activation of a specific set of genes, different from each mono-microbial infection. When we compared our total number of differentially regulated genes (2,423) to the DIRGs, we found that our study has identified 2,197 novel infection response genes, including 168 new core genes. Thus, our data offer a more comprehensive list of infection-responsive genes that is expanded both because of the sensitivity of RNA-seq technology over the previous microarrays and because of the broader diversity of bacteria used in our experiment.

To investigate the biological functions of our newly identified core response genes, we evaluated GO categories enriched in the core (Fig 3A and 3B). Upregulated core genes were primarily annotated with immune functions, such as Toll pathway and defense response to Gram-negative bacteria. This group also included genes involved in metabolism, including glycosaminoglycan metabolic process, carbohydrate metabolism, and metal ion transport. Additionally, core upregulated genes have a role in cellular and tissue processes, with genes acting in tissue repair, response to oxidative stress, cellular homeostasis, co-translational protein targeting to membrane, and protein targeting to ER (Fig 3A). The core downregulated genes were annotated with functions such as oxidation-reduction and starch and sucrose metabolism (Fig 3B). Core genes can be separated into two groups: genes regulated in response to live infections only and genes regulated in response to both live infections and heat-killed bacteria (Fig 3C). The 78 core genes that were also differentially expressed in the wound-only control and in the heat-killed bacteria control included genes coding for AMPs, PGRPs, Turandot (*Tot*) genes, and other classical targets of the Toll and Imd pathways [24]. Genes regulated only in response to live infection included key transcription factors of the immune system, such as *Rel* and *dl*, and were associated with biological processes such as metabolism, oxidation-reduction, regulation of iron ion transmembrane transport, and secretion. Altogether, these data indicate that heat-killed bacteria mostly trigger classically defined immune responses, while live infections regulate a set of additional biological processes that presumably reflect physiological interactions between the host and invading pathogen. These processes, including metabolic rewiring, response to stress and damage, cellular translation, and secretion, could act as physiological adaptations or buffers to the stress and damage imposed by infection.

The hypothesis that *D*. *melanogaster* has a distinct response to infection by Gram-positive (Lys-type PGN) versus Gram-negative (DAP-type PGN) bacteria dominated the field for most of the 1990s and 2000s [25]. To address this hypothesis, we characterized the transcriptional response to Gram-positive versus Gram-negative bacterial infection in our study. We found that 662 genes are regulated only by infection with Gram-positive bacteria, 851 genes are regulated only by Gram-negative infection, and 1,063 genes are regulated by infections with bacteria of both Gram types (Fig 3D). Of the 662 genes exclusively regulated by Gram-positive bacteria, only 20 (*Cyp309a1*, *daw*, *CG31326*, etc.) are upregulated and 8 are downregulated by all three Gram-positive bacteria. Similarly, amongst genes regulated specifically by Gram-negative bacteria, only 1 gene is upregulated (*AttD*) and no genes are downregulated in response to all 7 Gram-negative bacteria. Our data suggest that the stereotypical response to Gram-negative infection also occurs as a consequence of Gram-positive infection, such that there is no large cohort of genes responding exclusively to Gram-negative infection. To confirm this, we performed RT-qPCR on *Dpt* and *Drs* transcripts as a proxy for activity of the Imd and Toll pathways, respectively [7]. We found that infection by most of our 10 bacteria induced both pathways, although to significantly different levels (S6 Fig). Our results generally confirm the notion that the Toll pathway is more responsive to infection with Gram-positive (Lys-type PGN) bacteria and the Imd pathway is more reactive to infection with Gram-negative (DAP-type PGN) bacteria, but also make clear that the differences in pathway activation are quantitative and not qualitative or binary.

### Infection induces long-term changes in global host transcription

**Fig 4:**
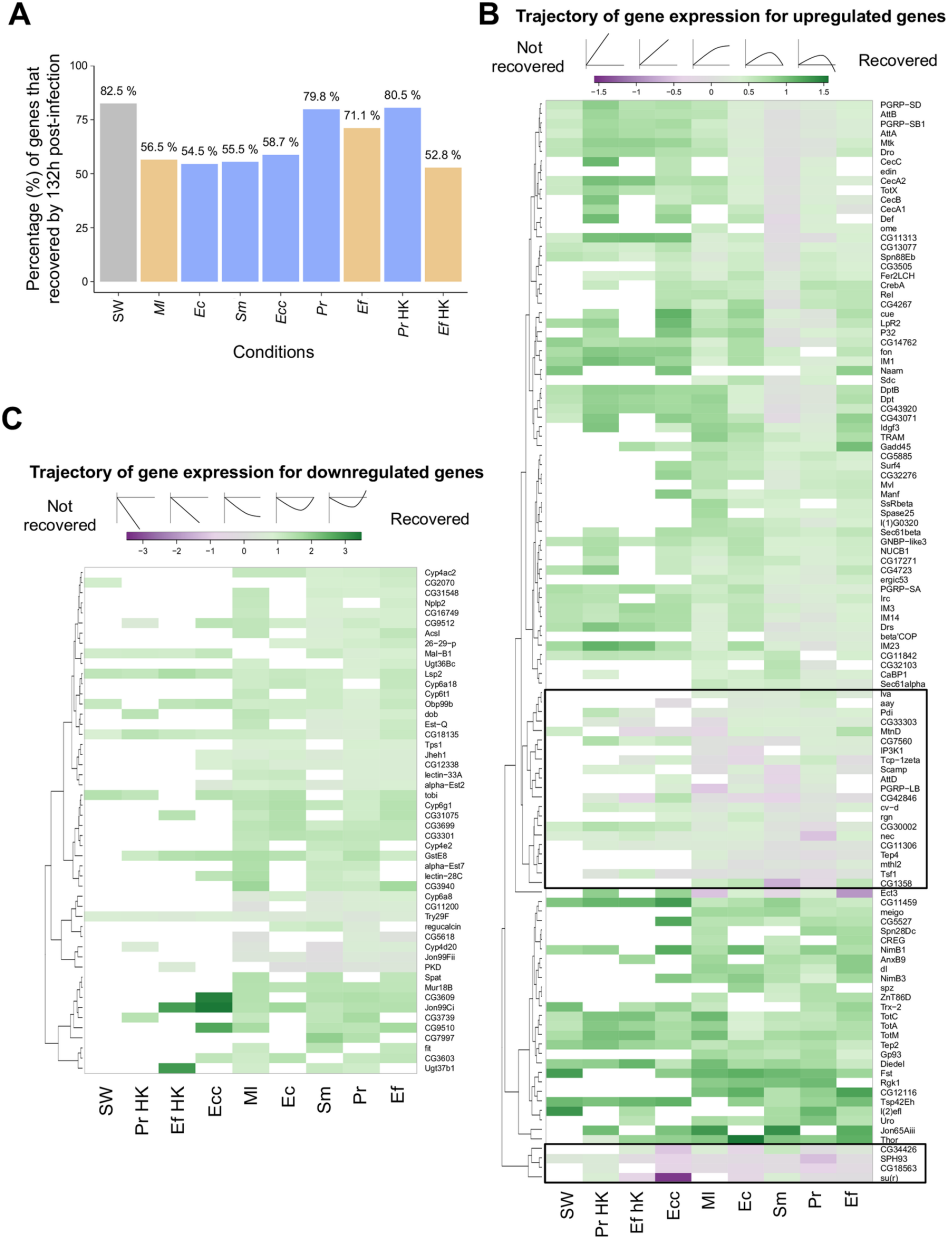
Bacterial infection elicits long-term changes in global host transcription. (A) Percentage of genes found to be differentially expressed at 12 and/or 36 h post-infection in a given condition that returned to basal levels of expression (recovery) by 132 h post-infection. (B) Gene expression trajectory of core upregulated genes. By 132 h post-infection, the expression level of core upregulated genes continued to increase (purple), plateaued (gray), or returned to basal, pre-infection levels (green) as indicated by the graphic above the color key. A black box encloses genes that did not recover in most infections (as denoted by the purple color). (C) Trajectory of gene expression for core downregulated genes. By the last time point (132 h), the transcript levels of core downregulated genes continued to decrease (purple), plateaued (gray), or returned to basal expression levels (green) as illustrated on the graphic above the color key. Genes that were not differentially regulated by a given condition are marked in white.

Since the bacteria belonging to the low and intermediate virulence categories do not kill all hosts, we followed the dynamics of gene expression in surviving hosts over several days. In particular, we aimed to contrast the sustained transcriptional response of flies that had cleared their infections to undetectable levels (i.e. after infection with *M*. *luteus* or *Ecc15*) to that of flies carrying chronic infections (i.e. *E*. *coli*, *S*. *marcescens* Type strain, *P*. *rettgeri* and *E*. *faecalis*). We hypothesized that persistent bacteria would continue to elicit a response from the host, which would be absent in flies that have cleared all bacteria. To test this idea, we determined whether genes “recover” from bacterial infection. We defined recovery in terms of gene expression: a gene that has recovered is differentially expressed at 12 and/or 36 h post-infection but returns to pre-infection levels by 132 h (5.5 days) after inoculation. We found that, on average, a minimum of 50% of the genes that are differentially regulated by each infection returned to basal levels by our last time point (Fig 4A), and this was the case even in hosts infected with persistent infections. The percentage of genes that fully recovered was substantially higher in moderately virulent infections (*P*. *rettgeri*: 79.8% and *E*. *faecalis*: 71.1%) than in benign infections (*E*. *coli*: 54.5% and *S*. *marcescens* Type: 55.5%), perhaps in part as a consequence of the higher number of genes induced upon infection with these bacteria (Fig 4A). Surprisingly, we also observed that only 56.5% and 58.7% of genes recovered in *M*. *luteus* and *Ecc15* infections, respectively, even though the majority of hosts (≥85%) survive these infections and the bacteria are eliminated (i.e. their levels fall below our detection threshold) within two days. These results demonstrate the complexities of the transcriptional response to infection. While there can be a substantial lingering transcriptional effect in flies that successfully cleared an infection, a subset of differentially regulated genes may return to basal levels even in chronically infected flies that continue to carry bacteria.

Next, we evaluated how the core upregulated and downregulated genes change in expression level over time (Fig 4B and 4C). We quantified the degree of recovery for each gene by comparing the fold change in expression at 132 h after infection to the fold change at either 12 h or 36 h, whichever was the highest if the gene was upregulated or the lowest if the gene was downregulated. In general, sterile wound and challenge with heat-killed bacteria resulted in the regulation of fewer core genes than live infection, and most of these genes recovered to pre-infection expression levels by 132 h post-challenge (Fig 4B and 4C). Core genes induced by *Ecc15* and *M*. *luteus* showed similar kinetics, and most genes had recovered or were on their way to recovery by 132 h, suggesting that the core response is not sustained in the absence of these bacteria. In contrast, core genes induced by *S*. *marcescens* Type, *P*. *rettgeri*, and *E*. *faecalis* did not recover as much, in agreement with the idea that infections with persistent bacteria continuously stimulate the core response. This paradigm was, however, not true for downregulated genes, as most downregulated genes did recover or were in the process of recovery by 132 h regardless of which bacteria was used for infection. Interestingly, we noticed that a group of genes did not recover at all in most conditions but continued to be upregulated over time (boxed in Fig 4B). These included effector genes of the immune response (*AttD* and *Tep4*), regulators of iron homeostasis (*Tsf1* and *MtnD*), and negative regulators of the immune response (*PGRP-LB*, *nec*). Genes like *SPH93* and *su(r)* never returned to their basal expression levels in flies infected with *Ecc15* or *M*. *luteus*. Additionally, while the transcript levels of most antimicrobial peptide genes decreased over time, they never returned to basal, pre-infection levels, suggesting that the effect of infection lingers for several days after bacteria are eliminated.

### *CrebA* is regulated in the fat body upon infection by the Toll and Imd pathways

**Fig 5:**
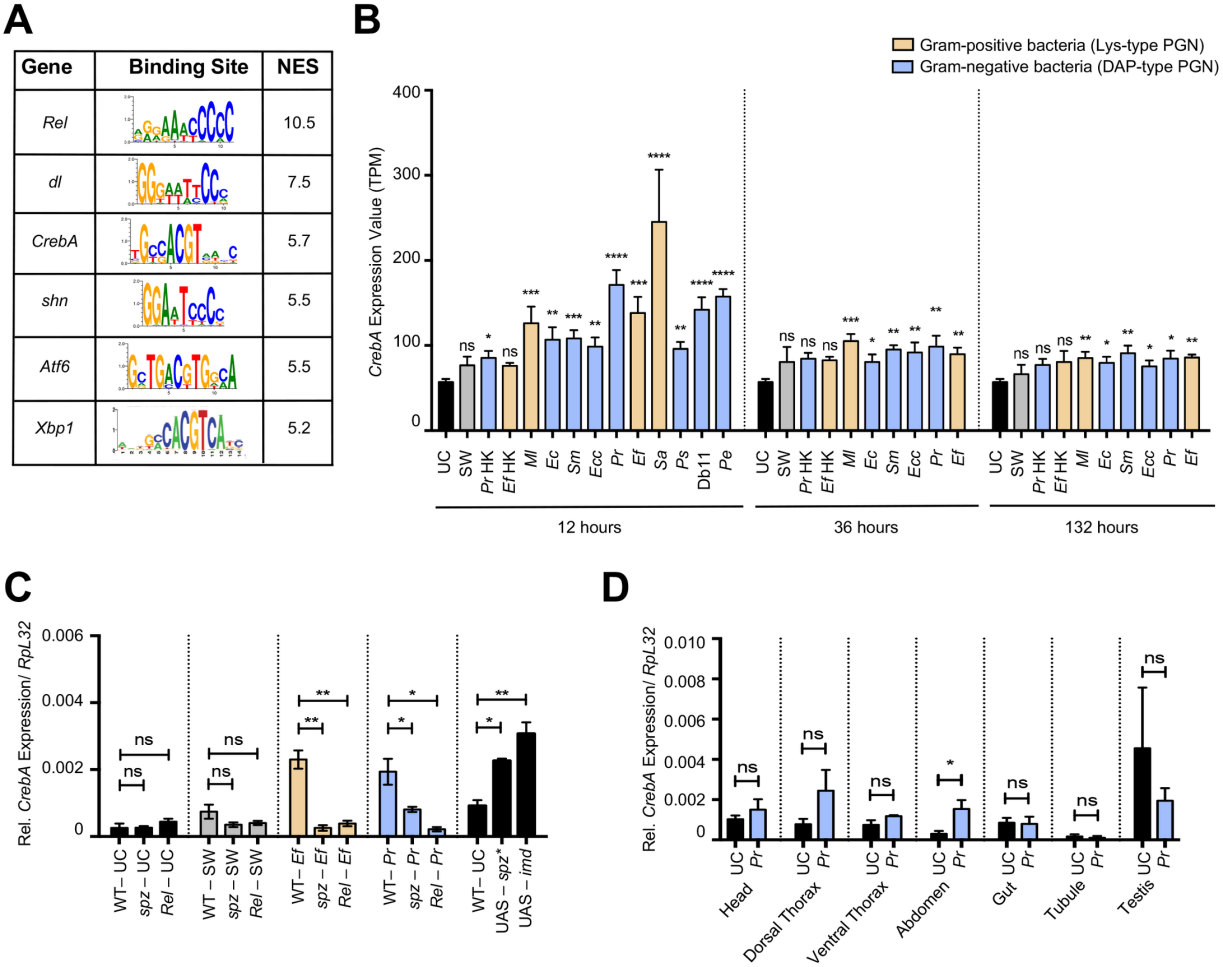
*CrebA* is a core transcription factor regulated by Toll and Imd in the fat body. (A) Subset of transcription factors whose predicted binding sites are enriched in the promoter regions of core upregulated genes. The table includes the transcription factors’ gene symbols, consensus binding sites, and their normalized enrichment scores (NES), which indicate the degree to which a binding site is overrepresented at the top of a ranked list of binding sites. (B) RNA-seq expression values in TPM (transcripts per million) of *CrebA* at 12, 36, and 132 h after infection with all 10 bacteria. (C) RT-qPCR of *CrebA* levels in *Rel*^E20^ and *spz*^rm7^ mutants and wildtype flies following: no challenge (UC), sterile wound (SW), infection with *E*. *faecalis* (*Ef*), and infection with *P*. *rettgeri* (*Pr*). In the last histogram, WT indicates wildtype flies given no challenge, UAS-spz* denotes *CrebA* expression in the absence of challenge when an activated form of Spz is ubiquitously overexpressed, and UAS-imd shows *CrebA* expression in flies that constitutively overexpress Imd in the absence of challenge. (D) RT-qPCR of *CrebA* levels in dissected organs and body parts (head, dorsal thorax, ventral thorax, abdomen, gut, Malpighian tubule, and testis) following infection with *P*. *rettgeri*. Mean values of at least three biological replicates are represented ±SE. *p<0.05 **p<0.01 ***p<0.001 ****p<0.0001 in a Student’s t-test.

Having identified a core transcriptional response to infection, we set out to find key regulators of that response. We used i-cisTarget to identify transcription factor binding motifs enriched in the regulatory regions of our core genes [26,27]. Using this approach, we found enrichment in putative binding sites for Relish (Rel), Dif/Dorsal, Schnurri (Shn), CrebA, Atf6, Xbp1, and Tbp in the regulatory regions of upregulated core genes (Fig 5A and S2 Table). Dif/Dorsal and Relish are the terminal transcription factors of the Toll and Imd pathways, respectively; therefore, finding enrichment for their predicted binding sites is in agreement with the central role that these pathways play in the immune response. Our data also agree with published reports showing that the TGF-beta pathway upstream of *shn* and the *Atf6* transcription factor are important to survive infection [28,29]. Transcription factor binding site enrichment analysis of the repressed genes revealed putative binding sites for the Lola and GATA transcription factors (S3 Table).

In addition to the i-cisTarget analysis, we searched for genes encoding transcription factors within our list of core upregulated genes. We identified 3 transcription factors in the defined core that are upregulated themselves by infection: *Rel*, *dorsal*, and *CrebA* (Fig 5B). Although *Dif* is required to activate Toll pathway signaling in response to bacterial infection in *Drosophila* adults and *dorsal* is not [30], we surprisingly found that *Dif* is not significantly upregulated in response to any of the 10 bacteria tested.

*CrebA* is the single *Drosophila* member of the Creb3-like family of transcription factors [31]. We found the predicted DNA motif bound by CrebA (TGCCACGT, see Fig 5A for position weight matrix [32]) in 71 genes upregulated by infection, including 18 upregulated core genes (S7A Fig). *CrebA* is itself significantly induced upon infection by all 10 bacteria (Figs 3A and 5B). To validate our RNA-seq results on *CrebA* expression, we infected a new group of flies with *P*. *rettgeri* and *E*. *faecalis* and measured *CrebA* transcript levels at 12 h post-inoculation. In agreement with our RNA-seq data, we confirmed that *CrebA* expression is upregulated in response to infection with *P*. *rettgeri* (p = 0.0026) and *E*. *faecalis* (p = 0.0147) (S7B Fig). These results demonstrate that *CrebA* is a transcription factor induced by infection and is potentially a key regulator of the core response.

To identify the molecular mechanisms that control *CrebA* transcription in response to infection, we scanned 2 kb upstream and 2 kb downstream of the *CrebA* transcription start site for potential transcription factor binding sites using MatInspector (Genomatix) [33]. Within this region, we found an enrichment of putative binding sites corresponding to the transcription factors Dif/Dorsal and Relish. There were 12 predicted Relish binding sites, 16 predicted Dif binding sites, and 13 predicted Dorsal binding sites flanking the *CrebA* gene, suggesting that immune pathways may induce the expression of *CrebA* (S7C Fig). To confirm regulation of *CrebA* by the Toll and Imd pathways, we quantified *CrebA* expression by RT-qPCR 12 h after infection with *P*. *rettgeri* and *E*. *faecalis* in wildtype (WT) flies, flies deficient for the Imd pathway (*Rel*^E20^), and flies deficient for the Toll pathway (*spz*^rm7^) (Fig 5C). *CrebA* expression was significantly reduced in both *Rel*^E20^ (p = 0.0456 for *P*. *rettgeri* and p = 0.0020 for *E*. *faecalis*) and *spz*^rm7^ (p = 0.0118 for *P*. *rettgeri* and p = 0.0026 for *E*. *faecalis*) mutants relative to wildtype controls, indicating that both the Imd and Toll pathways contribute to infection-induced *CrebA* upregulation. We then tested whether activation of the Imd or Toll pathway is sufficient to upregulate the level of *CrebA* expression in the absence of infection. Using the temperature-sensitive *UAS/Gal4/Gal80*^*ts*^ gene expression system to ubiquitously drive Imd or an active form of Spz (Spz*), we stimulated Imd and Toll pathway activity in adult flies [34,35]. Transgenic activation of either the Imd or Toll pathway in the absence of infection was sufficient to significantly increase *CrebA* transcript levels in *D*. *melanogaster* adults (p = 0.0114 for UAS-spz* and p = 0.0062 for UAS-imd) (Fig 5C). Altogether, our results demonstrate that the Imd and Toll pathways are both necessary and sufficient to regulate *CrebA* transcription upon infection.

In order to identify the tissue(s) and/or organ(s) within the fly that upregulate *CrebA* expression upon bacterial challenge, we infected wildtype flies with *P*. *rettgeri* and dissected out the following tissues and body parts at 12 h post-infection: head, dorsal thorax (including wings and heart), ventral thorax (including legs), digestive tract (crop, midgut, and hindgut), Malpighian tubules, testes, and abdomen (abdominal fat body). The abdomen was the only tissue that exhibited significant upregulation of *CrebA* as determined by RT-qPCR (p = 0.0315), suggesting that *CrebA* may be regulated in the fat body upon infection (Fig 5D). We therefore knocked down *CrebA* expression by RNAi (via 3 independent RNAi constructs) using 2 separate fat body drivers, *c564-Gal4* and *Lpp-Gal4*, and quantified *CrebA* expression by RT-qPCR in whole flies 12 h after infection with *P*. *rettgeri*. The combination of 2 *CrebA* RNAi constructs (B and C) with the drivers fully prevented *CrebA* induction upon infection with *P*. *rettgeri*. In the case of the third RNAi construct (A), *CrebA* was significantly upregulated by infection with *P*. *rettgeri* (p = 0.0002), but the induction was significantly lower (p = 0.0442) than the expression level observed in infected wildtype samples (S7D Fig). These data indicate that the cells of the fat body represent the primary site of *CrebA* induction. In sum, our data suggest that the Toll and Imd pathways regulate the expression of *CrebA* in the fat body in response to infection.

### *CrebA* is required to survive infection and promotes tolerance of infection

**Fig 6:**
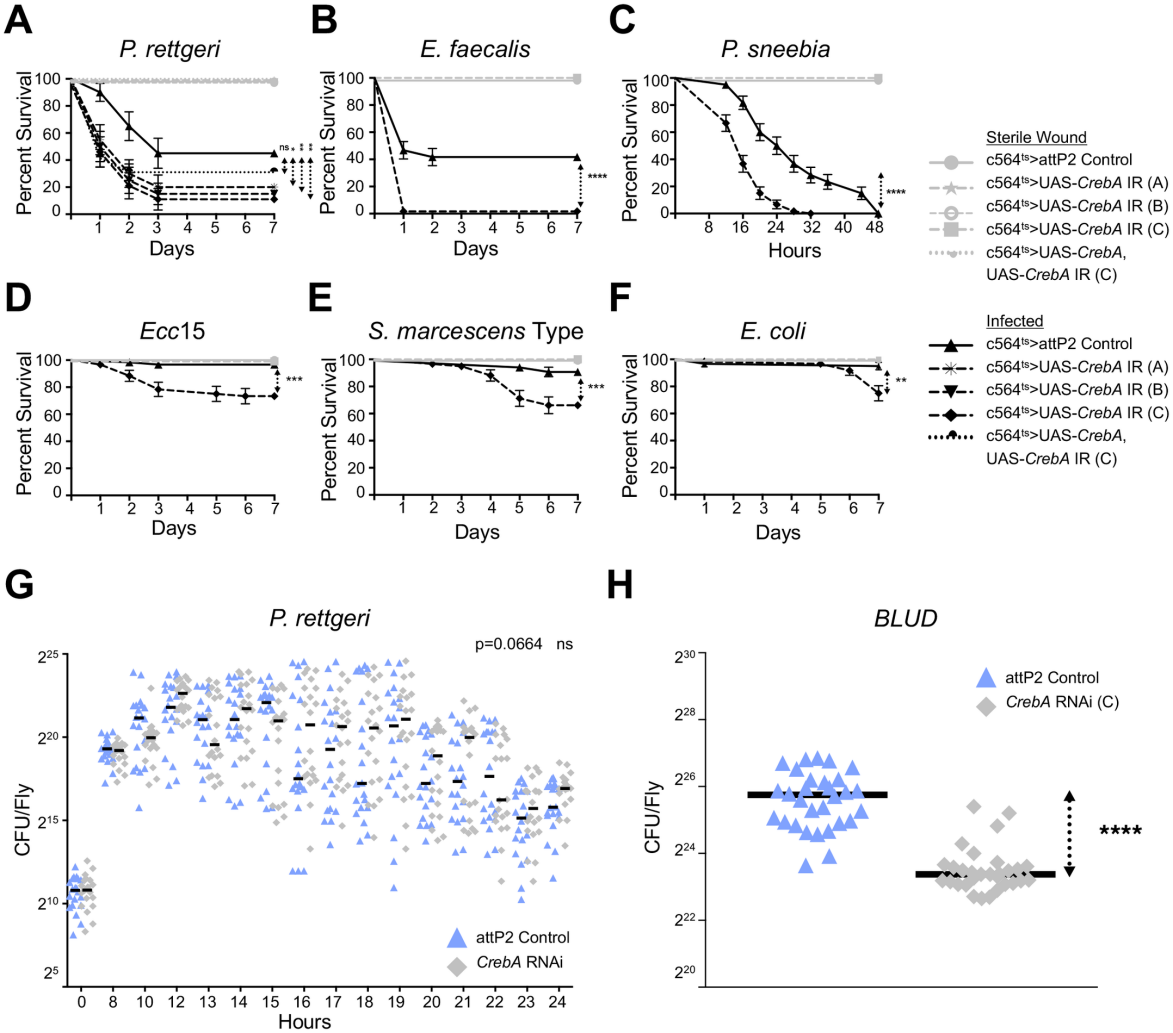
*CrebA* promotes infection tolerance. Survival curves over 7 days (or 48 h in the case of *P*. *sneebia*) following infection of flies whose expression of *CrebA* is blocked with RNAi. *UAS-CrebA IR* (A), (B), and (C) indicate three distinct RNAi constructs that target *CrebA* transcripts. *UAS-CrebA*, *UAS-CrebA* IR refers to flies simultaneously co-expressing a *CrebA* RNAi and a *CrebA* overexpression construct. *attP2* is the background genotype control, in which *CrebA* is fully expressed. Knockdowns were driven in the fat body and hemocytes using a conditional c564 temperature sensitive driver. The curves represent the average percent survival ±SE of three biological replicates. *p<0.05 **p<0.01 ***p<0.001 ****p<0.0001 in a Log-rank test. Infections were performed with (A) *P*. *rettgeri*. (B) *E*. *faecalis*. (C) *P*. *sneebia*. (D) *Ecc15*. (E) *S*. *marcescens* Type. (F) *E*. *coli*. (G) Bacterial load time course of control flies and flies expressing *CrebA* RNAi in the fat body following infection with *P*. *rettgeri*. (H) *P*. *rettgeri* bacterial load upon death (*BLUD*) of wildtype controls and flies with *CrebA* expression knocked down by RNAi in the fat body. Three repeats are graphed together, with each symbol representing an individual fly’s number of colony forming units (CFU). Horizontal lines represent median values for each condition. ****p<0.0001 in a Student’s t-test.

We next asked whether *CrebA* is required for the host to survive infection. Since strong loss-of-function *CrebA* mutants are embryonic lethal, we tested the role of *CrebA* in response to infection by knocking it down in the fat body of adult flies using 3 independent RNAi constructs expressed under the control of the *c564-Gal4* driver (*Gal80*^*ts*^*; c564-Gal4 > UAS-CrebA-IR*) and, separately, the *Lpp-Gal4* driver (*Gal80*^*ts*^*; Lpp-Gal4 > UAS-CrebA-IR*) [36]. Because the *c564-Gal4* driver expresses strongly in both the fat body and hemocytes, we additionally tested the requirement for *CrebA* in the response to infection in hemocytes (*Hml-Gal4 > UAS-CrebA-IR)*. All *CrebA* fat body knockdown flies exhibited increased susceptibility to systemic infection with *P*. *rettgeri* (p<0.0001) (Fig 6A and S8A Fig), while hemocyte-specific knockdown did not lead to any significant increase in mortality (S8B Fig). When *CrebA* was knocked down in the fat body, nearly 100% of the flies died, and most of the death occurred during the first 24 h following infection. In contrast, almost 50% of control flies survived the infection for at least 7 days (Fig 6A and S8A Fig). To confirm that the survival phenotype observed in *CrebA* RNAi flies upon infection was solely due to loss of *CrebA* expression, we co-expressed a *CrebA* RNAi construct and a *CrebA* overexpression construct in flies (*Gal80*^*ts*^*; c564-Gal4 > UAS-CrebA*, *UAS-CrebA-IR*) and infected them with *P*. *rettgeri*. We observed no significant difference between the survival of infected control flies and that of infected flies co-expressing both the RNAi and overexpression constructs, indicating that changes in *CrebA* expression are uniquely responsible for the lowered survival phenotype observed (Fig 6A). We also infected *CrebA* RNAi flies with *E*. *faecalis* and found that *CrebA* RNAi flies were remarkably more susceptible to infection when compared to control flies (p<0.0001) (Fig 6B). In addition, *CrebA* RNAi flies died at a significantly faster rate than control flies when inoculated with *P*. *sneebia* (p<0.0001) (Fig 6C). Finally, infection with *Ecc15*, *S*. *marcescens* Type, and *E*. *coli* also killed more flies with *CrebA* expression blocked in the fat body than controls (p = 0.0013 for *Ecc15*, p = 0.0004 for *S*. *marcescens* Type, and p = 0.0028 for *E*. *coli*) (Fig 6D–6F). None of these latter three infections were lethal to wildtype control flies, but approximately 30% of *CrebA*-deficient flies succumbed to infection. Collectively, our results demonstrate that *CrebA* is generally required to survive bacterial infection.

To test whether the *CrebA* survival phenotype is due to a failure to control bacterial proliferation (a resistance defect) or a decrease in the ability to withstand infection (a tolerance defect), we monitored bacterial load in individual *CrebA* RNAi and control flies following *P*. *rettgeri* infection [37]. We focused our sampling on 1–2 h intervals over the first 24 h of infection, as this is the time when most of the *CrebA*-deficient flies succumbed. We did not find a significant difference in bacterial load between wildtype and *CrebA* knockdown flies at any measured time point (p = 0.0664), indicating that *CrebA* RNAi flies are able to control bacterial load similarly to control flies (Fig 6G). To corroborate these results, we quantified bacterial load following infection with *P*. *rettgeri* in flies where *CrebA* was knocked down by a different RNAi construct and in flies co-expressing a *CrebA* RNAi construct and a *CrebA* overexpression construct (*Gal80*^*ts*^*; c564-Gal4 > UAS-CrebA*, *UAS-CrebA-IR*). Again, we did not observe any significant difference in bacterial load between wildtype and *CrebA* knockdown flies (p = 0.3208) or between wildtype and *CrebA* rescue flies (p = 0.3030) (S8C Fig). To evaluate whether *CrebA* knockdown flies are less resistant to other pathogens, we measured bacterial load in individual flies following *E*. *faecalis* or *Ecc15* infection. In agreement with the results of our *P*. *rettgeri* experiments, we did not find a significant difference between wildtype and *CrebA*-deficient flies at the time points sampled (p = 0.4204 for *E*. *faecalis* and p = 0.7253 for *Ecc15*) (S8D and S8E Fig), suggesting that *CrebA* knockdown flies do not have a defect in resistance to infection.

We previously demonstrated that flies die at a stereotypical and narrowly distributed bacterial load, the bacterial load upon death (*BLUD*), which represents the maximum quantity of bacteria that a fly can sustain while alive [38]. We therefore sought to determine whether *CrebA* RNAi flies have a lower *BLUD*, which would indicate a reduced tolerance of infection. We quantified the bacterial load of individual flies within 15 minutes of their death and found that *CrebA* RNAi flies died carrying a significantly lower bacterial load than control flies (p<0.0001) (Fig 6H). These data demonstrate that while *CrebA*-deficient flies control bacterial growth normally, they are more likely to die from infection, and they die at a lower bacterial load than wildtype flies. Therefore, the transcription factor *CrebA* acts to promote tolerance of infection.

### Loss of *CrebA* alters the expression of secretory genes during infection

**Fig 7:**
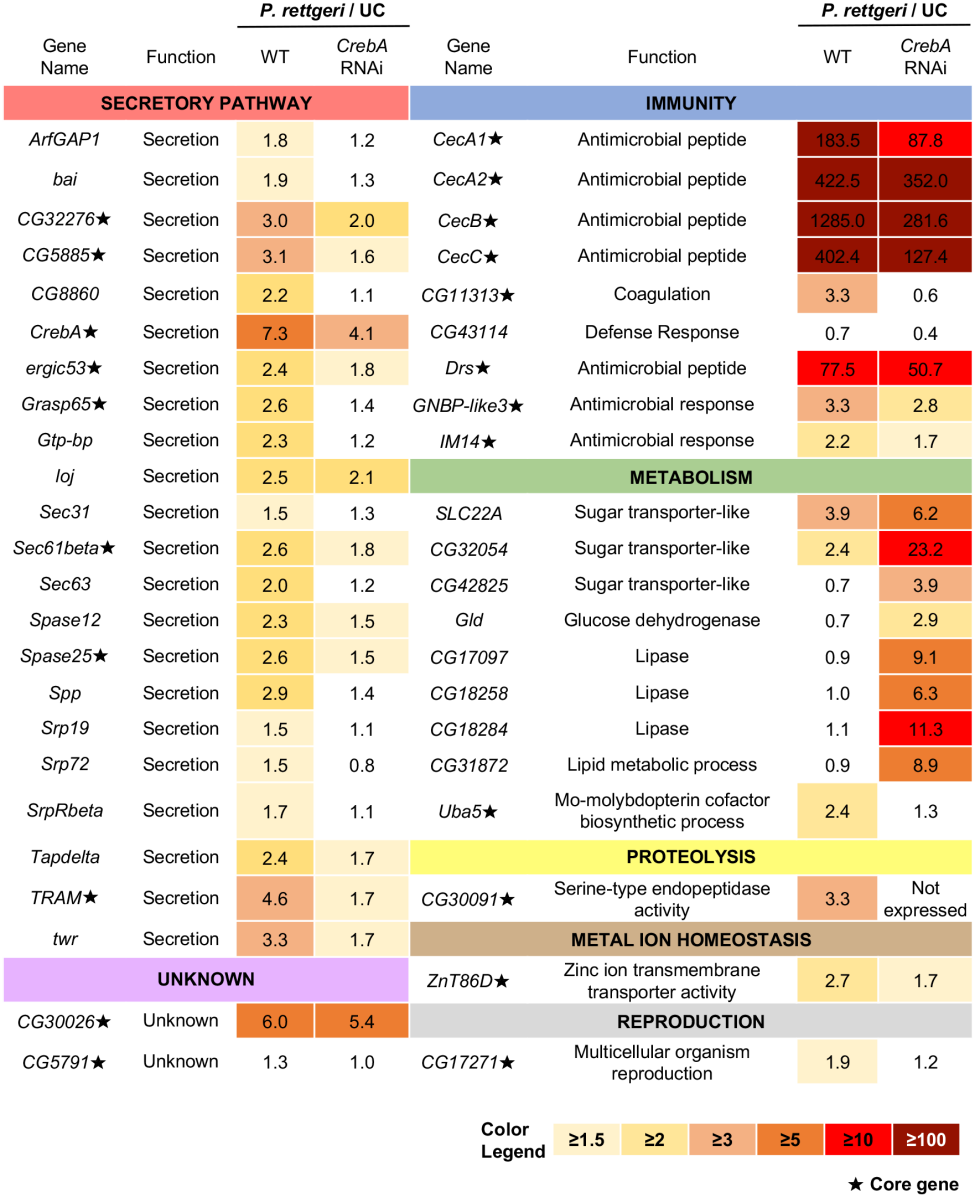
*CrebA* regulates the expression of secretory pathway genes upon infection. Select list of 45 genes whose expression significantly changes in infected *CrebA* RNAi fat body samples compared to infected control samples. Gene symbols, functions, and fold enrichment of expression with infection (*P*. *rettgeri*/unchallenged) are indicated. Core genes are highlighted with a ★ symbol.

In order to identify the complete set of genes directly and indirectly regulated by *CrebA* upon infection, we performed RNA-seq on the fat bodies of wildtype flies and flies in which we knocked down *CrebA* in the fat body. We collected samples from both genotypes in unchallenged conditions and 12 h after infection with *P*. *rettgeri*. In total, we found that only 104 genes were downregulated in *CrebA* knockdown fat bodies compared to wildtype fat bodies following infection (S5 Table). These genes were associated with GO categories such as protein targeting to the ER, signal peptide processing, protein localization to the ER, and antibacterial humoral responses. Antimicrobial peptide genes of the *Cecropin* gene family (*CecA1*, *Cec*A2, *CecB*, and *Cec*C) showed partially reduced induction when *CrebA* expression was disrupted. Nevertheless, they were still induced to extremely high levels (>200-fold) in *CrebA* knockdown fat bodies (Fig 7). Other antimicrobial peptide genes, such as *Dpt*, *Drs*, *Def*, and *AttC*, were expressed at similar levels in *CrebA* knockdown fat bodies compared to wildtype fat bodies, results corroborated by RT-qPCR analysis (S9A–S9D Fig). In contrast, a number of genes including sugar transporters and multiple lipases were upregulated upon infection in fat bodies deficient for *CrebA* but not in wildtype fat bodies. These data suggest that *CrebA* regulates immune, metabolic, and cellular functions during infection.

Previously, Fox and colleagues demonstrated that *CrebA* acts in the *Drosophila* embryo as a direct regulator of secretory capacity and is both necessary and sufficient to activate the expression of many secretory pathway component genes [32]. We therefore asked whether *CrebA* controls secretion-related genes upon infection in the adult fat body. We found that the expression level of 32 secretion-related genes significantly increased upon infection with *P*. *rettgeri* in wildtype samples. However, the induction of these secretion-related genes was significantly lower (p<0.05) in *CrebA* RNAi fat body samples compared to wildtype fat body controls, a result that agrees with the findings of Fox et al. (Fig 7 and S5 Table). These 32 secretion-related genes we identified included core response genes that are central components of the cell’s secretory machinery, including *TRAM*, *ergic53*, *Sec61β*, and *Spase25* (Fig 7). Using a separate set of samples from those of the RNA-seq, we further confirmed these findings by measuring *TRAM*, *ergic53*, *Sec61β*, and *Spase25* transcript levels by RT-qPCR in the fat bodies of flies infected with *P*. *rettgeri* at 12 h post-infection (S9E–S9H Fig). These four genes were significantly upregulated following infection with *P*. *rettgeri* in wildtype samples. However, we were not able to detect a significant increase in the levels of *TRAM*, *ergic53*, and *Sec61β* in *CrebA* RNAi fat bodies upon infection. The expression level of *Spase25* was significantly induced by infection with *P*. *rettgeri* even when *CrebA* expression was inhibited by RNAi in the fat body (p<0.05), but the induction was significantly lower (p<0.001) than the expression level observed in infected wildtype samples (S9H Fig). In sum, our data suggest that *CrebA* could act to regulate an increase in secretory capacity upon infection.

### *CrebA* deficiency leads to ER stress upon bacterial challenge

**Fig 8:**
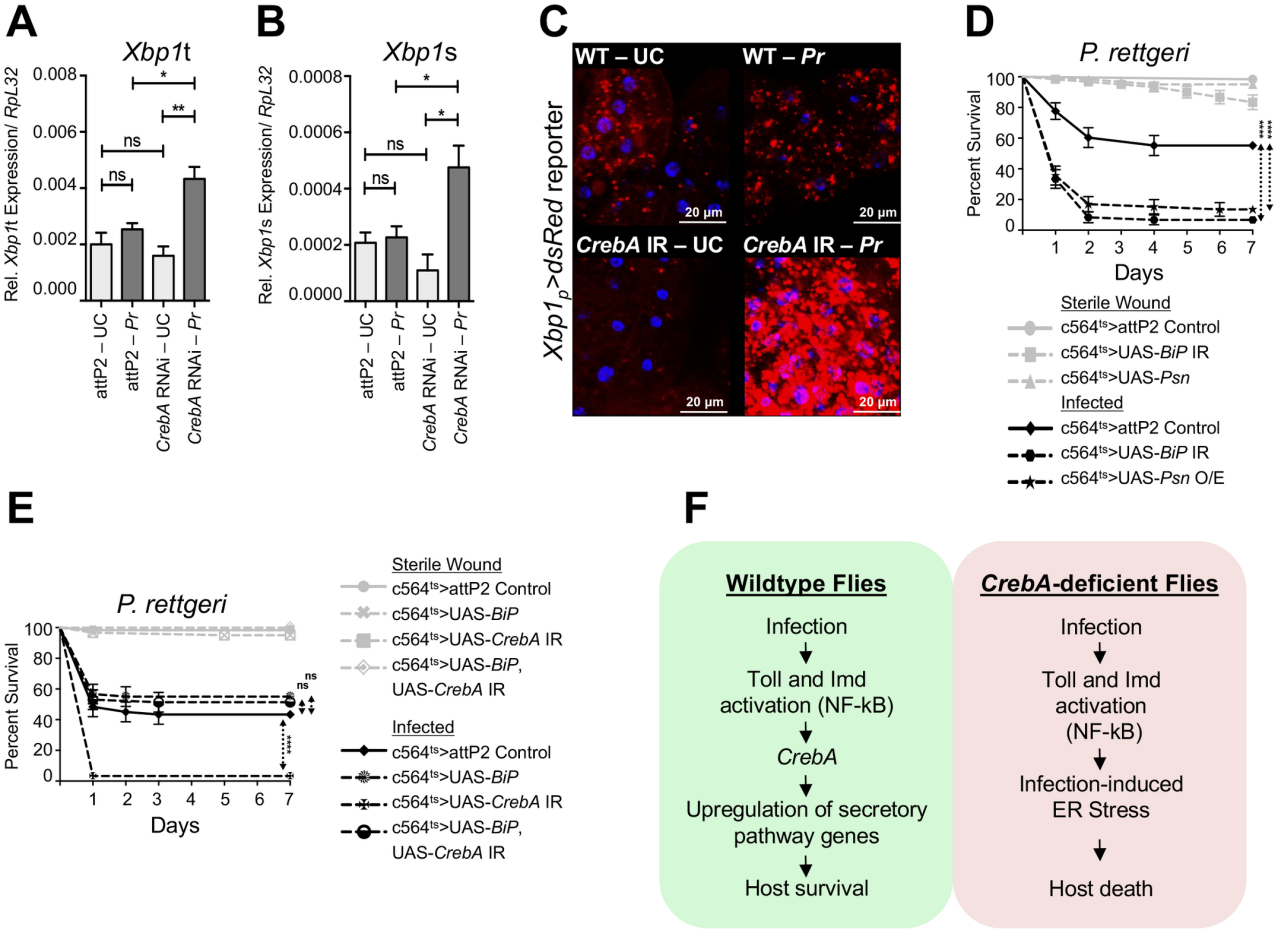
Loss of *CrebA* triggers ER stress during infection. RT-qPCR of *Xbp1*t (unspliced, inactive) (A) and *Xbp1*s (spliced, active) (B) levels in the fat bodies of *CrebA* RNAi and wildtype flies in unchallenged and infected (*P*. *rettgeri*) conditions. An increase in the spliced form of *Xbp1* (*Xbp1*s) is a sign of ER stress. Mean values of three repeats are represented ±SE. *p<0.05 **p<0.01 in a Student’s t-test. (C) Fat bodies from the *Xbp1*_*p*_*>dsRed* reporter crossed to *CrebA* RNAi or wildtype flies in unchallenged (UC) and infected conditions (*Pr*). (D) Survival curves of flies with genetically-induced ER stress (*Psn* overexpression or *BiP* RNAi) in the fat body in unchallenged and infected conditions. (E) Survival curves over 7 days of flies co-expressing both the *CrebA* RNAi and *BiP* overexpression constructs in their fat bodies following infection with *P*. *rettgeri*. The curves represent the average percent survival ±SE of three biological replicates. ****p<0.0001 in a Log-rank test. (F) Upon infection, activation of the Toll and Imd pathways in the fat body transcriptionally upregulates the expression of the transcription factor *CrebA*. In turn, *CrebA* upregulates the expression of secretory pathway genes. In absence of *CrebA*, a failure to upregulate secretion machinery genes leads to infection-induced ER stress, followed by host death.

Since our data suggested that *CrebA* may promote an increase in secretory capacity in the fat body upon infection, we hypothesized that loss of *CrebA* expression could lead to altered protein secretion or defects in protein transport to the membrane. Accumulation of unfolded proteins or a decrease in protein secretion triggers endoplasmic reticulum (ER) stress, which in turn induces stereotypical pathways to limit the stress imposed on the cell. These pathways include IRE1α/XBP1-, PERK/ATF4-, and ATF6-mediated responses termed the unfolded protein response (UPR) [39,40]. Upon sensing of ER stress, *Xbp1* mRNA undergoes alternative splicing via IRE1α; *Xbp1* splicing is thus considered to be a marker of ER stress and of the activation of UPR [41,42]. To investigate whether loss of *CrebA* could trigger ER stress in fat body cells upon infection, we quantified the expression levels of both *Xbp1t* (total) and *Xbp1s* (spliced) in abdomens of wildtype and *CrebA*-knockdown flies under both unchallenged and infected conditions (Fig 8A and 8B). *Xbp1s* levels did not change upon infection in wildtype samples or differ between wildtype and *CrebA* RNAi samples in the absence of infection. However, *Xbp1s* levels spiked dramatically in *CrebA* RNAi fat body samples (p = 0.0289) (Fig 8B) after infection, indicating that loss of *CrebA* upon bacterial challenge triggers ER stress in the fat body. Our data also revealed that *Xbp1t* expression was significantly higher in *CrebA* knockdown samples compared to wildtype samples following infection (p = 0.0144) (Fig 8A). This result is in agreement with a previous study that suggested *Xbp1s* regulates *Xbp1* transcription [43]. To determine whether ER stress is induced in fat body cells directly, we labelled fat body cells *in vivo* by expressing a *dsRed* reporter under the control of the *Xbp1* regulatory sequence [44]. In agreement with our RT-qPCR experiments, we found that bacterial challenge did not induce *dsRed* expression in wildtype samples. However, infected *CrebA* RNAi fat body cells consistently expressed higher levels of *dsRed* compared to all other controls (Fig 8C). These results demonstrate that *CrebA* expression prevents the occurrence of ER stress in the fat body upon infection.

We next asked whether the failure of *CrebA*-deficient flies to prevent ER stress following infection could explain their increased susceptibility to bacterial challenge. To test this, we genetically induced ER stress in *Drosophila* fat bodies either by overexpression of *Psn* (*Gal80*^*ts*^*; c564-Gal4 > UAS-Psn*), which disrupts calcium homeostasis, or by knockdown of *BiP* (*Gal80*^*ts*^*; c564-Gal4 > UAS-BiP-IR*), a regulatory protein of the unfolded protein response [45,46]. Inducing ER stress in the fat body during infection made the flies more susceptible to *P*. *rettgeri* infection, phenocopying the result observed with *CrebA* knockdown flies (p<0.0001 for both constructs) (Fig 8D). Since the increased susceptibility of *CrebA* RNAi flies to infection stemmed from a tolerance defect (Fig 6G and 6H and S8C–S8E Fig), we sought to determine whether the increase in mortality observed in *BiP* RNAi and *Psn* overexpression flies following infection is also due to a tolerance deficiency. We monitored bacterial load in individual *BiP* RNAi and *Psn* overexpression flies following challenge with *P*. *rettgeri*. We did not observe a significant difference in bacterial load between wildtype and *BiP*-knockdown flies (p = 0.0624) or between wildtype and *Psn* overexpression flies (p = 0.6462) (S10A Fig). Quantification of bacterial load upon death (*BLUD*) following *P*. *rettgeri* infection in *BiP* RNAi flies showed that *BiP*-deficient flies perish carrying a significantly lower bacterial load than wildtype flies (p<0.0001) (S10B Fig). Altogether, our data indicate that induction of fat body ER stress during infection decreases fly survival by lowering host tolerance of infection.

Having demonstrated that *CrebA*-deficient flies experience fat body ER stress upon bacterial challenge and that flies with genetically induced fat body ER stress display increased mortality without a concomitant change in bacterial load following infection, thus phenocopying *CrebA*-deficient flies, we subsequently asked whether alleviating ER stress in *CrebA*-deficient flies could rescue the *CrebA* survival phenotype. To test this, we overexpressed *BiP* in fat body cells in which *CrebA* was knocked down by RNAi (*Gal80*^*ts*^*; c564-Gal4 > UAS-CrebA-IR*, *UAS-BiP*). Previous work has shown that overexpression of *BiP* can ameliorate ER stress [47]. While overexpression of *BiP* alone did not alter host survival during infection, expression of *BiP* in *CrebA* RNAi flies rescued fly survival upon challenge with *P*. *rettgeri* (Fig 8E). We observed no significant difference between the survival of infected control flies and that of infected flies co-expressing both the *CrebA* RNAi and *BiP* overexpression constructs (p = 0.2786). These data indicate that reducing ER stress is sufficient to rescue the survival phenotype of *CrebA*-deficient flies during bacterial challenge. Excessive and prolonged ER stress can lead to apoptosis [48]. Therefore, we investigated whether *CrebA* RNAi flies are more susceptible to infection due to an increase in fat body cell apoptosis. We blocked apoptosis by overexpressing the apoptosis inhibitor P35 in the fat body of *CrebA*-knockdown flies (*Gal80*^*ts*^*; c564-Gal4 > UAS-CrebA-IR*, *UAS-P35*) [49]. Expression of P35 in *CrebA* RNAi flies did not rescue the *CrebA* survival phenotype upon infection (S10C Fig), indicating that an increase in apoptosis is unlikely to explain the *CrebA* susceptibility defect. Collectively, our results show that *CrebA* is required in the fat body to prevent excessive and deleterious levels of ER stress upon infection.

## Discussion

### Bacteria trigger diverse and unique host responses

In this study, we have characterized the transcriptomic response of *Drosophila* to a wide range of bacterial infections. We found that the response to infection can involve up to 2,423 genes, or 13.7% of the genome. This is a considerably greater number of genes than what has been previously reported in similar transcriptomic studies [6,8]. As the response to infection was highly specific to each bacterium, the larger number of genes we identified is likely a consequence of having included more bacterial species in our experiment than previous studies. Likewise, we anticipate that future studies using different species of bacteria could further increase the number of genes found to be involved in the host response to infection. Our data clearly establish that while the core response to infection is narrow and conserved, every bacterium additionally triggers a very specific transcriptional response that reflects its unique interaction with host physiology.

At first, this high level of specificity may seem contrary to the traditional vision of the innate immune response. Early studies defined the innate immune system as generic, and the specificity of the *Drosophila* immune response was considered as a dichotomous activation of the Toll pathway by Gram-positive bacteria (Lys-type peptidoglycan) or the Imd pathway by Gram-negative bacteria (DAP-type peptidoglycan) [5,25]. Our data show that the host response to infection goes beyond the activation of the Toll and Imd pathways, with each bacterium also modulating host cell biology, metabolism, and stress responses in a microbe-specific manner. Although we did find that the type of bacterial peptidoglycan is a key factor shaping the response, we also found that each bacterium activates both the Toll and Imd pathways to quantitatively different levels, consistent with previous reports suggesting a much more complex coordination of the immune response [50–53]. Activation of the Toll and Imd pathways depends on recognition of microbe-associated molecular patterns (MAMPs) and detection of damage-associated molecular patterns (DAMPs), suggesting that virulent bacteria could activate the Toll and Imd pathways to a higher degree [13,14]. However, we did not find a clear correlation between the virulence level of the bacterium or bacterial load sustained and the degree to which the canonical immune response is activated. In sum, our results support the notion that the response to infection comprises more than simple activation of immune functions, but instead is a function of precise physiological interactions between host and microbe.

### Identification of a core response to infection

Although the response to infection appears to be largely specific, we identified a core set of genes that are regulated by infection with most bacteria. Induced genes include the classical targets of the Toll and Imd pathways, such as antimicrobial peptides and immune effectors (TEPs and IMs). However, genes involved in cell and tissue biology (translation, secretion, cell division) were also upregulated by the majority of infection conditions, possibly indicating a response to the stress imposed by infection. On the other hand, genes involved in metabolism (protease activity, oxidation-reduction, glucose metabolism, respiration), as well as digestive enzymes (e.g. the maltase cluster), were downregulated, suggesting a complete reshaping of host metabolism during infection [6]. It is tempting to speculate that the majority of core genes that do not fall under the immunity category could be part of a tolerance core response. Although the subject of tolerance mechanisms has attracted a lot of interest in recent years, identifying the genes and processes that define tolerance has remained somewhat elusive [54,55]. Further characterization of the core genes identified here may shed light on universal tolerance mechanisms.

The idea of a core response to infection has also been explored in other organisms. In *Caenorhabditis elegans*, for example, a study using four different pathogens to assay the transcriptional response to infection found that the core of the response included genes involved in proteolysis, cell death, and stress responses [56]. Comparative transcriptomics work in the honey bee, *Apis mellifera*, also revealed a core set of genes utilized in response to distinct pathogens, including genes involved in immunity, stress responses, and tissue repair [57]. In *Danio rerio*, immunity, metabolism, and cell killing have been implicated in host defense [58]. Collectively, these results and ours indicate that there is considerable overlap in the core response to infection across species, and that this consistency extends beyond classical immune sensing and signaling. Having a well-defined core response to infection in *Drosophila* will allow future studies to quantitatively assess differences in how distinct pathogens induce the core, as well as test the relative importance of various elements of the core in promoting resistance to and tolerance of infection.

A surprisingly high proportion (~40%) of the core response to infection was induced only by live microbes, but was not stimulated by challenge with heat-killed bacteria. One possibility is that MAMPs, such as peptidoglycan, are partially, if not fully, degraded at the sampled time points, obscuring our ability to appreciate the full extent of the response to MAMPs. An alternative explanation is that almost half of the core response to infection is a reaction to microbial activity, rather than just to the presence of MAMPs. This latter model involves the detection of the host’s own DAMPs upon infection [12]. For example, bacterial growth and secretion of toxins can inflict damage to host tissues, leading to the generation of DAMPs, such as actin, proteases, and elastases [13,14,59]. In turn, DAMPs can activate the Toll, Imd, and JAK-STAT pathways, which may trigger higher levels of signaling in these pathways beyond that which is induced by the detection of MAMPs [13,14,16,59]. Higher degrees of activation in these pathways could then translate into the induction of a larger set of target genes, which could partially account for the ~40% of core genes that are uniquely induced by live infections.

### Bacterial infection triggers long term changes in host transcription

Interestingly, our study found that gene expression levels do not always reflect the changes in bacterial load during the course of infection. In chronically infected flies, we found that most genes downregulated at 12 h post-infection had returned to baseline expression levels by 132 h after infection. Likewise, many of the induced genes also decreased in expression or returned to basal levels even while flies still harbored bacteria. It is possible that the injury inflicted to systemically infected flies generates a complex early response, which is resolved at later time points. However, we note that injury alone did not generally trigger the downregulation of genes observed in live infections. An alternative explanation is that the bacteria have entered into a less aggressive state in the late stages of infection, persisting but with a reduced impact on the physiology of their host. Yet another hypothesis is that the host’s initial response to infection is broad-spectrum and disproportionately strong, with the proactive goal of suppressing all bacteria before they can establish a highly detrimental infection. In this scenario, a subdued infection can be controlled with more nuance at later stages [22]. Finally, it is also possible that the percentage of recovered genes following infection with moderately virulent bacteria is overestimated because the RNA-seq is performed on pools of flies that may have distinct individual fates upon infection, and therefore distinct transcriptional kinetics. We have previously shown that flies infected with these same bacteria either die with a high bacterial load or survive with a low-level, persistent infection [38]. The individual flies at the 12 h RNA-seq data point comprise flies destined for both outcomes, but only persistently infected flies are sampled at the 132 h time point after mortality has occurred. If flies fated to die induce genes that are not triggered in flies destined to survive, those genes may appear to be upregulated in the pooled 12 h RNA sample that contains a mix of flies destined for both outcomes. Likewise, those same genes will appear to have returned to baseline levels at the 132 h time point when just chronically infected flies are sampled, creating the false impression that they have recovered. Future work is required to evaluate these hypotheses and to provide insight into how the complex dynamics of gene expression relate to changes in pathogen burden [60].

We also observed seemingly long-term alterations to the transcription of some core response genes, even in the case of infections with bacteria, such as *M*. *luteus* and *Ecc15*, that are reduced to undetectable levels or cleared by the host. For example, the expression of several antimicrobial peptide genes (*Drs*, *Dro*, and *AttB*) as well as other effector molecules (*IM4* and *IM3*) never returned to basal levels, even multiple days after elimination of the infection. Such sustained reactions could provide long-lasting benefits in an environment with high risk of infection. Moreover, it should perhaps be considered that the baseline expression levels of these genes in laboratory-reared *Drosophila* are artificially low because of aseptic maintenance conditions as compared to those in natural environments.

### The transcription factor *CrebA* prevents infection-induced ER stress

Among our core response genes, we identified *CrebA* as a key transcription factor that promotes host tolerance to infection. CrebA is the single *Drosophila* member of the Creb3-like family of transcription factors, which includes five different proteins in mammals: Creb3/Luman, Creb3L1/Oasis, Creb3L2/BBF2H7, Creb3L3/CrebH, and Creb3L4/Creb4 [31]. A recent study demonstrated that *CrebA* is a master regulator of secretory capacity, capable of regulating the expression of the general machinery required in all cells for secretion [32]. *Drosophila CrebA* appears to have the same functional role as its mammalian counterparts. Exogenous expression of mammalian liver-specific *CrebH* caused upregulation of genes involved in secretory capacity and increased secretion of specific cargos [31]. Moreover, each of the five human CREB3 factors is capable of activating secretory pathway genes in *Drosophila*, dependent upon their shared ATB (Adjacent To bZip) domain [31]. In agreement with the function of CrebA and CREB3 proteins described in the literature, our study finds that *CrebA* regulates a rapid, infection-induced increase in the expression of secretory pathway genes in the fat body, an organ analogous to the liver and adipose tissues of mammals. Finally, it has been shown that proinflammatory cytokines act to increase the transcription of *CrebH*, and that CrebH becomes activated in response to ER stress [61]. Our data demonstrate that the two principal immune pathways in *Drosophila*, the Toll and Imd pathways, upregulate the expression of *CrebA* in response to bacterial challenge and that loss of *CrebA* in the fat body triggers ER stress upon infection. Collectively, the functions of mammalian *CrebH* as a regulator of secretory homeostasis under stress bear a striking resemblance to the role that we have attributed to *Drosophila CrebA* after bacterial challenge, suggesting that *CrebH* could have a similar role in mammals during infection.

CREB proteins are activated by phosphorylation from diverse kinases, including PKA and Ca2+/calmodulin-dependent protein kinases on the Serine 133 residue [62]. CrebA does not contain a PKA consensus phosphorylation site, and its transcriptional activity is only slightly enhanced by cAMP [36]. Rather, we found that the Toll and Imd pathways are both necessary and sufficient to regulate *CrebA* expression in the fat body. Loss of *CrebA* leads to ER stress, further aggravating the physiological strains of infection. However, a lack of *CrebA* in unchallenged conditions does not lead to the induction of ER stress. We therefore propose a model in which the Toll and Imd pathways act early to upregulate *CrebA* in order to adapt the fat body cells for infection, thus preventing ER stress that would otherwise be triggered by the response to infection [63] (Fig 8F). This interpretation would suggest that immune activation generates a massive and rapid increase in translation [64] and secretion in response to infection, and thus triggers cellular stress in the fat body. In that context, the Toll and Imd pathways would proactively induce expression of *CrebA* to prevent some of the stress that comes from their own activation.

Lastly, *CrebA* knockdown flies are more likely to die from infection yet they show no increase in pathogen burden. This demonstrates that *CrebA* is required for tolerance of infection [65,66]. Considering that ER stress is induced upon infection in the absence of *CrebA*, our data suggest that *CrebA* is a tolerance gene that helps mitigate the stress imposed by the host response to infection. Fast induction of *CrebA* by the immune system upon infection can therefore be interpreted as an active tolerance mechanism that is generally required to survive bacterial infection.

## Materials and methods

### Whole fly RNA-seq infections

Whole fly RNA-seq experiments were performed using wildtype strain *Canton S* flies. Flies were raised on standard yeast-cornmeal-sucrose medium (50 g baker’s yeast, 60 g cornmeal, 40 g sucrose, 7 g agar, 26.5 mL Moldex (10%), and 12 mL Acid Mix solution (4.2% phosphoric acid, 41.8% propionic acid) per 1L of deionized H_2_O) at 24°C and maintained at that temperature for the duration of the experiment. Individual males were infected with one of the ten experimental bacteria 5 to 8 days after eclosion from the pupal case. Control flies that were sterilely wounded or inoculated with heat-killed bacteria were handled equivalently. Flies were pin-pricked to generate septic injury. We standardized the initial inoculation dose across all bacteria to deliver ~3,000 colony-forming units (CFU) per fly. The following bacteria (from overnight cultures) were used: *Micrococcus luteus* (*A*_600_ = 100), *Escherichia coli* (*A*_600_ = 100), *Serratia marcescens* Type (*A*_600_ = 1), *Ecc15* (*A*_600_ = 1), *Providencia rettgeri* (*A*_600_ = 1), *Enterococcus faecalis* (*A*_600_ = 1), *Staphylococcus aureus* (*A*_600_ = 1), *Providencia sneebia* (*A*_600_ = 1), *Serratia marcescens* Db11 (*A*_600_ = 1), and *Pseudomonas entomophila* (*A*_600_ = 1). Three sets of controls were included in the experiment: unchallenged and uninjured flies, sterilely wounded flies, and challenge with either heat-killed *P*. *rettgeri* or heat-killed *E*. *faecalis*. For every control and bacterial infection, with the exception of the 4 highly virulent infections, 20 flies were collected at 12 h, 36 h, and 132 h post-infection. For the 4 highly virulent bacteria, only the 12 h sample was collected because the majority of the flies had died before the later time points. Additionally, 20 unchallenged, uninjured flies were also collected at time 0 h as an extra control. Each sample of 20 flies was homogenized, and total RNA was isolated using a modified TRizol extraction protocol (Life Technologies). All experiments were done in triplicate. The same methodology was employed for the RNA-seq experiment focused specifically on the fat body. Data can be downloaded from NCBI Sequence Read Archive with accession number SRP127794.

### 3’-end RNA-seq library construction and sequencing

Following RNA extraction, the 3’end RNA-seq libraries were prepared using QuantSeq 3’ mRNA-Seq Library Prep kit (Lexogen). The sample quality was evaluated before and after the library preparation using Fragment Analyzer (Advanced Analytical). Libraries were sequenced on two lanes of the Illumina Nextseq 500 platform using standard protocols for 75bp single-end read sequencing at the Cornell Life Sciences Sequencing Core.

### Read processing, alignment, counts estimation, and PCA

On average, 6 million reads per sample were sequenced at their 3’ termini. This is roughly equivalent in sensitivity to 20x coverage depth under a conventional random-priming RNA-seq method. Raw reads were first evaluated by fastqc for quality control (http://www.bioinformatics.bbsrc.ac.uk/projects/fastqc, version 0.11.3) and were then trimmed using Trimmomatic version 0.32 [67]. Trimmed reads were mapped to the *D*. *melanogaster* reference transcriptome, which was constructed with the *D*. *melanogaster* reference genome (version 6.80) using STAR RNA-seq aligner version 2.4.1a [68]. Read depth at each transcript was then calculated using htseq (version 0.6.1) [69]. Principal Component Analysis and extraction of the PC1/PC2 genes were performed by custom R scripts (available upon request).

### Differential expression, functional category and pathway, and transcription factor enrichment

The software edgeR version 3.10.5 was used to call the genes that are differentially expressed among treatments [70]. Nine samples of unchallenged flies matching the 3 different time points post-infection (12 h, 36 h, and 132 h) were collapsed into a single control once it was determined that their transcriptomic profiles were very similar. Library sizes were normalized using a trimmed mean of M-values (TMM) approach implemented in edgeR. Genes with low counts (count-per-million < 1.2) were filtered out prior to differential expression analysis. Genes were considered statistically differentially expressed if they were differentially expressed between unchallenged condition and an infection condition of choice at the 5% false discovery rate (FDR). A fold-change cutoff was not applied to the data. Each gene was also evaluated for the number of infection conditions in which it was differentially regulated, where an “infection condition” refers to the transcriptomic profile in response to any of the live infections or controls at any point post-infection. Heatmaps were generated and clustering was performed using custom R scripts. Gene Ontology and KEGG pathway enrichment analysis was performed using the DAVID bioinformatics resource [71] and PANTHER [72]. The p-values from these analyses were corrected using the Benjamini and Hochberg procedure [73] with the FDR threshold set to 0.05. The search for putative transcription factor binding sites was performed using i-cisTarget under the default parameter values [26,27].

### Defining the kinetics of the transcriptional response to infection

For each gene under each infection condition, an expression path was assigned based on the series of inferred induction or repression of infection relative to unchallenged controls at each successive time point. Genes that were significantly induced or repressed at 12 or 36 h but then returned to basal expression levels were deemed to have “recovered”. To quantify the degree of recovery for each gene, the level of fold change at 132 h after infection was compared to the fold change in expression at either 12 h or 36 h using custom R scripts (available upon request). The genes that were significantly induced (or repressed) at 12 h and then significantly repressed (or induced) at 36 h relative to the unchallenged conditions (1% of the genes) were excluded, as were genes that never changed expression in any of the time points.

### Fly strains and crosses for *CrebA* experiments

Subsequent to the initial RNA-seq experiment, genetic manipulations of *CrebA* expression were performed. Flies for all of these experiments were reared at 18°C or 24°C. The *Rel*^*E20*^ and *spz*^*rm7*^ stocks have been previously described [74,75]. For manipulation of *CrebA* expression level, we used the *UAS/Gal4* gene expression system in combination with *Gal80*^*ts*^ to restrict the expression of the constructs specifically to the adult stage. Male flies were collected 5 to 8 days after eclosion from the pupal case and then shifted to 29°C for an additional 8 days prior to any experiments. We used the following genotypes: 1. *c564-Gal4*; *tub-Gal80*^*ts*^, *UAS-GFP* 2. *Lpp-Gal4*; *tub-Gal80*^*ts*^, *UAS-GFP* 3. *c564-Gal4*; *tub-Gal80*^*ts*^, *UAS-CrebA-IR* 4. *UAS-imd* 5. UAS-spz* 6. *UAS-P35* 7. *UAS-Psn* (Bloomington 8305) 8. *UAS-BiP-IR* (Bloomington 32402) 9. *UAS-BiP* (Bloomington 5843) 10. TRiP control line *attP2* (Bloomington 36303) 11. TRiP control line *attP40* (Bloomington 36304) 12. *Xbp1*_*p*_*>dsRed* 13–15. *UAS-CrebA-IR* (Bloomington 42562 (A), 31900 (B) and 27648 (C)).

### Survival experiments

Infection was done via septic pinprick to the thorax. After inoculation, death was recorded daily, and flies were transferred to fresh vials every 3 days. All experiments were performed at least 3 times. Statistical significance was determined using a Log-rank (Mantel-Cox) test.

### Quantification of bacterial CFUs

At specified time points following infection, flies were individually homogenized by bead beating in 500 μl of sterile PBS using a tissue homogenizer (OPS Diagnostics). Dilutions of the homogenate were plated onto LB agar using a WASP II autoplate spiral plater (Microbiology International), incubated overnight at 29°C, and the CFUs were counted. All experiments were performed at least 3 times. Results were analyzed using a two-way (genotype and time) ANOVA in Prism (GraphPad Prism V7.0a, GraphPad Software, La Jolla, CA, USA).

### RT-qPCR

For all experiments utilizing RT-qPCR, total RNA was extracted from pools of 20 flies using a standard TRIzol (Invitrogen) extraction. RNA samples were treated with DNase (Promega), and cDNA was generated using murine leukemia virus reverse transcriptase (MLV-RT) (Promega). qPCR was performed using the SSO Advanced SYBR green kit (Bio-Rad) in a Bio-Rad CFX-Connect instrument. Data represent the relative ratio between the Ct value of the target gene and that of the reference gene *RpL32* (also known as *Rp49*). Mean values of at least three biological replicates are represented ±SE. Data were normalized and then analyzed using an unpaired t-test in Prism (GraphPad Prism V7.0a; GraphPad Software, La Jolla, CA, USA). The primer sequences used in this study are available in S6 Table.

### Fat body imaging

In some experiments, fat bodies were visualized microscopically. For these experiments, *Drosophila* abdomens were dissected and fixed in a 4% paraformaldehyde in 1X PBS solution for 45 minutes and washed 3 times with 0.1% Triton-X in PBS. DNA was stained in 1:50,000 DAPI (Sigma-Aldrich) in PBS and 0.1% Triton-X for 45 minutes. Samples were then washed three times in PBS and mounted in antifadent medium (Citifluor AF1). Imaging was performed on a Zeiss LSM 700 fluorescent/confocal inverted microscope.

## Supporting information

S1 Fig 10 bacteria cause different mortalities in *Drosophila*. Survival curves (in %) over time of control and infected *Canton S* flies. Three biological replicates are graphed independently for each condition. Treatments are as follows: (A) Unchallenged. (B) Sterile wound. (C) *Micrococcus luteus*. (D) *Enterococcus faecalis*. (E) *Staphylococcus aureus*. (F) *Escherichia coli*. (G) *Serratia marcescens* Type strain. (H) *Pectinobacterium* (formerly *Erwinia*) *carotovora Ecc15*. (I) *Providencia rettgeri*. (J) *Providencia sneebia*. (K) *Serratia marcescens* strain Db11. (L) *Pseudomonas entomophila*.
(TIF)

S2 Fig 10 bacteria differ in their rate of growth in the fly. Bacterial load time courses of infected *Canton S* flies over 132 h following infection. Three biological repeats are graphed together, with each triangle representing the bacterial burden in an individually sampled fly. (A) *M*. *luteus*. (B) *E*. *faecalis*. (C) *S*. *aureus*. (D) *E*. *coli*. (E) *S*. *marcescens* Type. (F) *Ecc15*. (G) *P*. *rettgeri*. (H) *P*. *sneebia*. (I) *S*. *marcescens* Db11. (J) *P*. *entomophila*. The symbol † denotes no flies were sampled because most, if not all, flies had succumbed by that time point. A number followed by the symbol Δ indicates the number of flies found to have no bacteria (flies that carry undetectable levels of bacteria or that have cleared the infection) at the specified time point.
(TIF)

S3 Fig Each bacterial infection induces a unique host response. (A) PCA plot showing the first two principal components of the entire dataset. Pink, orange, and red (warm) colors show infections with Gram-positive (Lys-type PGN) bacteria, while green, blue, and purple (cool) colors denote infections with Gram-negative (DAP-type PGN) bacteria. HK indicates stimulation with heat-killed bacteria. (B) Histogram of differentially upregulated (top) or downregulated (bottom) genes by the number of infection conditions in which a given gene was differentially expressed. (C) Percentage of genes that are uniquely upregulated (top) or downregulated (bottom) by each infection.
(TIF)

S4 Fig Individual infections differ in their ability to induce the Toll and Imd pathways and reshape host metabolism. Heatmap (log_2_ fold change) of top 100 genes that contribute the most to PC1 (A) and of top 100 genes that most contribute to PC2 (B). A gene was deemed to be regulated by the Toll or Imd pathways if the absence of the key genes in each pathway (*spz* for Toll and *Rel* for Imd) changed the expression level of said gene by 20% or more compared to the expression level of the gene in wildtype, as previously reported [7]. A color scale on the left side of each heatmap indicates whether each gene is regulated by Toll (T) or Imd (I). In the first column (T), genes regulated by the Toll pathway are marked in black, while genes not regulated by Toll are marked in beige. Similarly, in the second column (I), genes regulated by Imd are marked in black, while genes not regulated by Imd are marked in beige. Genes were marked in gray when no information was available about their regulation by Toll or Imd.
(TIF)

S5 Fig Overlap between genes responding to live infection, sterile wound, heat-killed bacteria, and the DIRGs. (A) Venn diagram showing the intersection between genes that are differentially regulated in response to at least one live infection, sterile wound, and challenge with heat-killed bacteria. (B) Venn diagram depicting the overlap between the previously described *Drosophila* Immune-Regulated Genes (DIRGs) [6], core genes differentially regulated in response to live infection, and genes differentially expressed in response to heat-killed bacteria. (C) Venn diagram illustrating the overlay between genes differentially regulated in response to *M*. *luteus* infection and, separately, *E*. *coli* infection in the present study and the DIRGs, which were previously identified from infection with a mixed cocktail of *E*. *coli* and *M*. *luteus* [6].
(TIF)

S6 Fig 10 bacteria induce different expression levels of antimicrobial peptide genes. RT-qPCR measuring (A) *Diptericin* and (B) *Drosomycin* expression levels in control and infected *Canton S* flies at 12, 36, and 132 h post-infection. These samples are separate biological replicates, distinct from those used in the RNA-seq experiment. Mean values of three biological repeats are represented ±SE. *p<0.05 **p<0.01 ***p<0.001 ****p<0.0001 in a Student’s t-test.
(TIF)

S7 Fig *CrebA* is a target of Toll and Imd and a putative regulator of core genes in the fat body. (A) Heatmap showing the expression levels (log_2_ fold change) of a select group of putative *CrebA* target genes found to be significantly upregulated by infection. Core genes (marked by a ★) and their functions are highlighted. (B) RT-qPCR validation of *CrebA* induction levels 12 h after infection with *P*. *rettgeri* (*Pr*) and *E*. *faecalis* (*Ef*) using samples distinct from those used in the RNA-seq. (C) Schematic of predicted Dif, Dorsal, and Relish binding sites on the *CrebA* promoter region (+/-2kb from the start site). (D) Whole fly RT-qPCR of flies with *CrebA* knockdown in the fat body following infection with *P*. *rettgeri*. *CrebA* RNAi (A), (B), and (C) denote three distinct RNAi constructs used to target *CrebA* mRNA. Mean values of three or more repeats are represented ±SE. *p<0.05 **p<0.01 ***p<0.001 in a Student’s t-test.
(TIF)

S8 Fig *CrebA* RNAi flies do not carry a larger bacterial burden than wildtype flies upon infection. (A) Survival curves over 7 days following *P*. *rettgeri* infection of flies whose expression of *CrebA* is blocked with RNAi specifically in the fat body with a second driver, *Lpp-Gal4* (*Gal80*^*ts*^*; Lpp-Gal4 > UAS-CrebA-IR*). *attP2* is the background genotype control, in which *CrebA* is fully expressed. (B) Survival of unchallenged and infected (*P*. *rettgeri*) control flies and flies expressing *CrebA* RNAi in hemocytes only (*Hml-Gal4* driver). The curves represent the average percent survival ±SE of three biological replicates. ****p<0.0001 in a Log-rank test. (C) Bacterial load time course of control flies, flies expressing a separate *CrebA* RNAi construct (construct B), and flies simultaneously co-expressing a *CrebA* RNAi and a *CrebA* overexpression construct in the fat body following infection with *P*. *rettgeri*. Bacterial load time course of *CrebA* knockdown and control flies after infection with (D) *E*. *faecalis* and (E) *Ecc15*. Three repeats are graphed together, with each symbol representing an individual fly’s number of colony forming units (CFU). Horizontal lines represent median values for each condition. A number followed by the symbol Δ (*attP2* control flies) or the symbol **♢** (*CrebA* RNAi flies) indicates the number of flies found to have no bacteria (flies that carry undetectable levels of bacteria or that have cleared the infection) at the specified time point.
(TIF)

S9 Fig *CrebA* regulates secretory capacity during infection. Expression level of predicted *CrebA* target genes in unchallenged (UC) or infected (*Pr*) conditions in control (*attP2*) and *CrebA* RNAi fat body samples. Assayed genes encoding antimicrobial peptides are (A) *Diptericin*, (B) *Drosomycin*, (C) *Defensin*, and (D) *Attacin C*. Surveyed genes encoding secretory factors are (E) *TRAM*, (F) *ergic53*, (G) *Sec61beta*, and (H) *Spase25*. Mean values of three biological replicates are represented ±SE. *p<0.05 **p<0.01 ***p<0.001 ****p<0.0001 in a Student’s t-test.
(TIF)

S10 Fig *CrebA* expression prevents ER stress upon infection. (A) Bacterial load time course of control flies and flies expressing *BiP* RNAi or *Psn* overexpression in the fat body following infection with *P*. *rettgeri*. (B) Bacterial load upon death (*BLUD*) following *P*. *rettgeri* infection of wildtype controls and flies with *BiP* expression knocked down by RNAi in the fat body. Three repeats are graphed together, with each symbol representing an individual fly’s number of colony forming units (CFU). Horizontal lines represent median values for each condition. ****p<0.0001 in a Student’s t-test. (C) Survival curves of flies co-expressing *CrebA* RNAi and the apoptosis inhibitor P35 in fat body cells. The curves represent the average percent survival ±SE of three biological replicates.
(TIF)

S1 Table Core upregulated and downregulated genes with functional annotations and the level of gene expression change at 12 h after infection with each bacterium (log_2_ fold change). (XLSX)

S2 Table Putative transcription factor binding sites enriched in core upregulated genes as ascertained by i-cisTarget. (XLSX)

S3 Table Putative transcription factor binding sites enriched in core downregulated genes as ascertained by i-cisTarget. (XLSX)

S4 Table Expression kinetics of core upregulated and downregulated genes. (XLSX)

S5 Table List of genes differentially regulated between infected wildtype fat bodies and infected *CrebA* RNAi fat bodies. (XLS)

S6 Table List of primers used in RT-qPCR experiments. (XLSX)
