# Supplementary figures and images for: Comparative transcriptomics reveals CrebA as a novel regulator of infection tolerance in D. melanogaster
Source: PLoS Pathog. 2018 Feb 2;14(2):e1006847. doi: 10.1371/journal.ppat.1006847 (PMC5812652; doi:10.1371/journal.ppat.1006847)

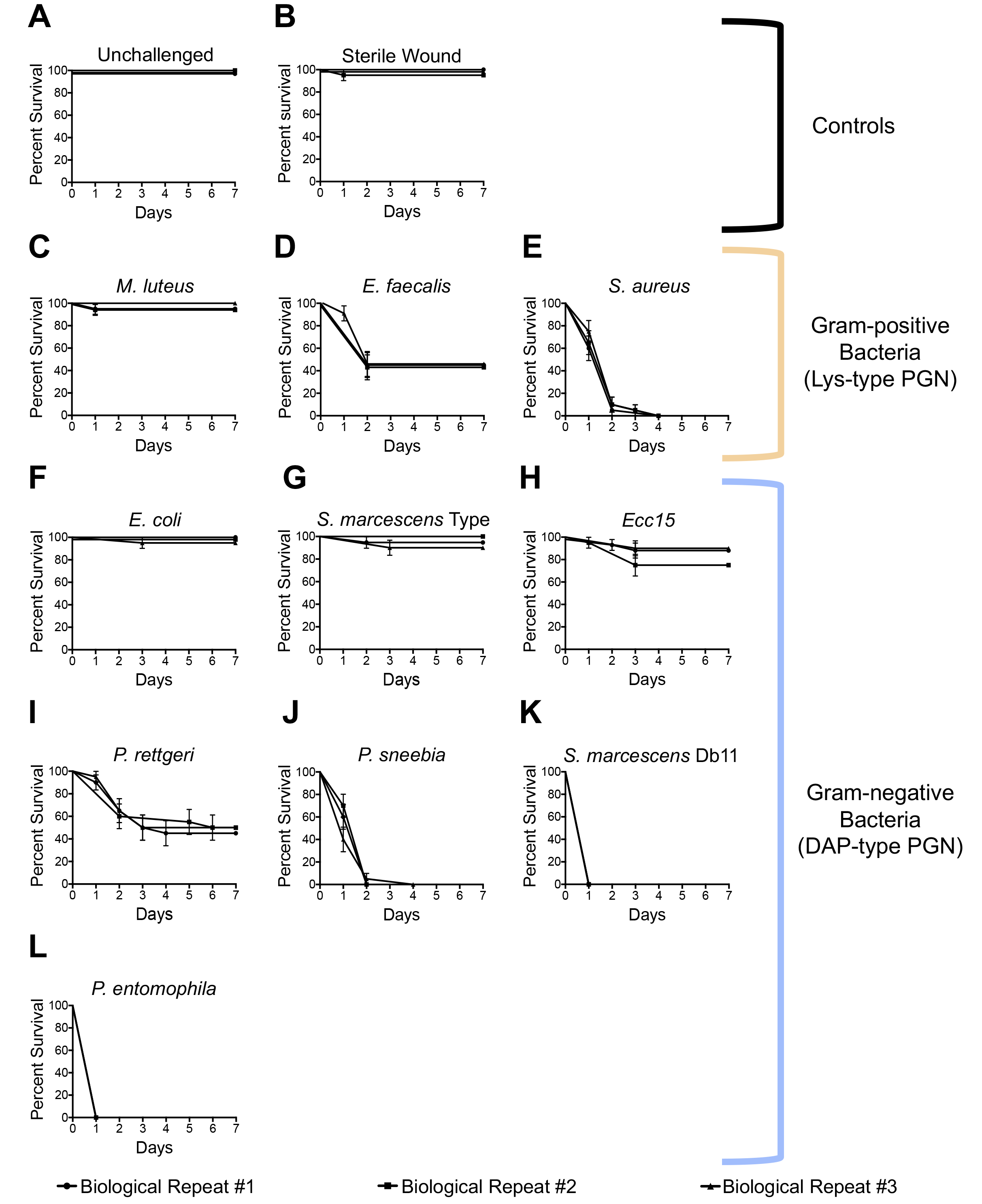

Supplement: S1 Fig — Survival curves (in %) over time of control and infected Canton S flies. Three biological replicates are graphed independently for each condition. Treatments are as follows: (A) Unchallenged. (B) Sterile wound. (C) Micrococcus luteus. (D) Enterococcus faecalis. (E) Staphylococcus aureus. (F) Escherichia coli. (G) Serratia marcescens Type strain. (H) Pectinobacterium (formerly Erwinia) carotovora Ecc15. (I) Providencia rettgeri. (J) Providencia sneebia. (K) Serratia marcescens strain Db11. (L) Pseudomonas entomophila. (TIF) [file ppat.1006847.s001.tif]

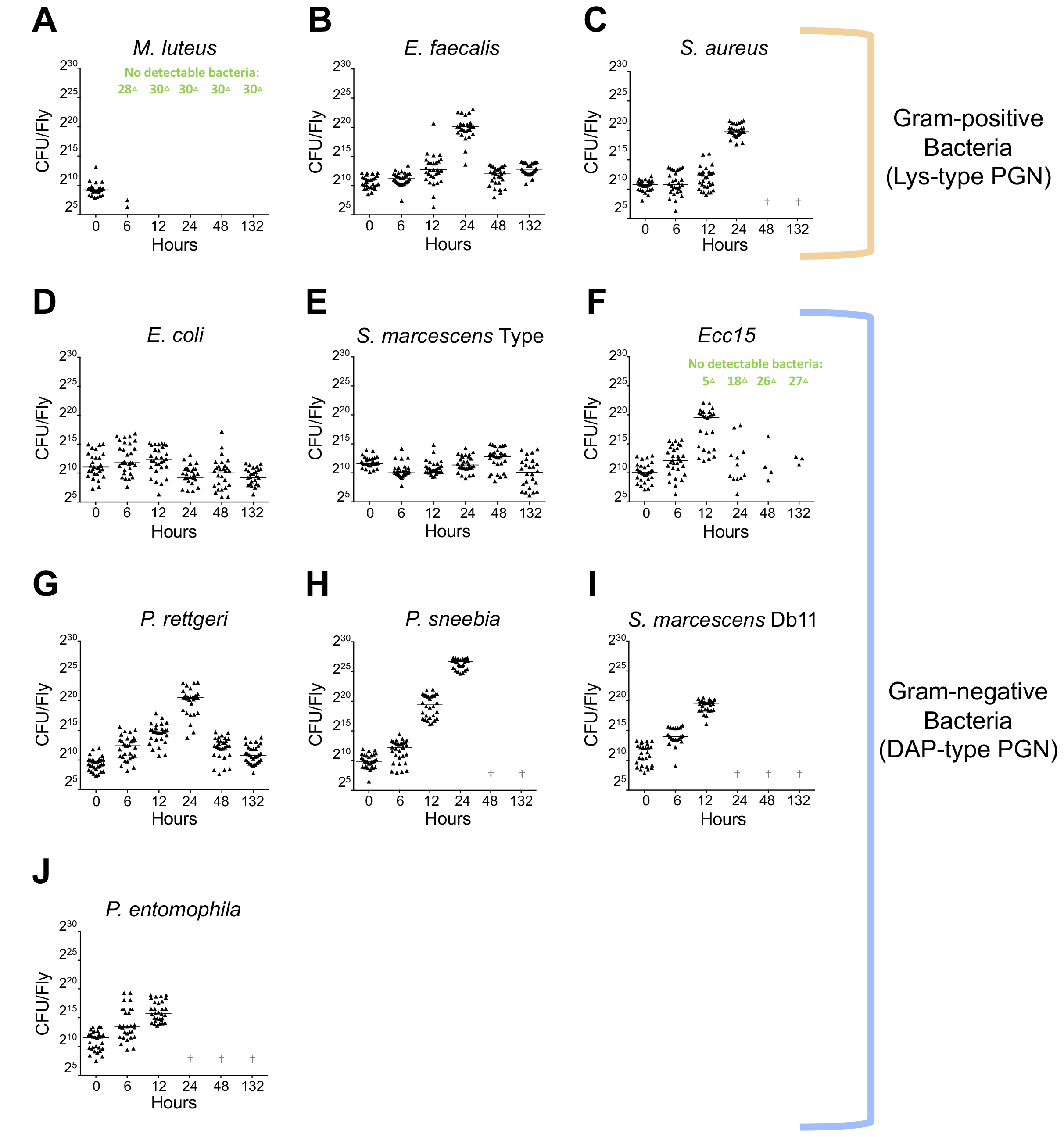

Supplement: S2 Fig — Bacterial load time courses of infected Canton S flies over 132 h following infection. Three biological repeats are graphed together, with each triangle representing the bacterial burden in an individually sampled fly. (A) M. luteus. (B) E. faecalis. (C) S. aureus. (D) E. coli. (E) S. marcescens Type. (F) Ecc15. (G) P. rettgeri. (H) P. sneebia. (I) S. marcescens Db11. (J) P. entomophila. The symbol † denotes no flies were sampled because most, if not all, flies had succumbed by that time point. A number followed by the symbol Δ indicates the number of flies found to have no bacteria (flies that carry undetectable levels of bacteria or that have cleared the infection) at the specified time point. (TIF) [file ppat.1006847.s002.tif]

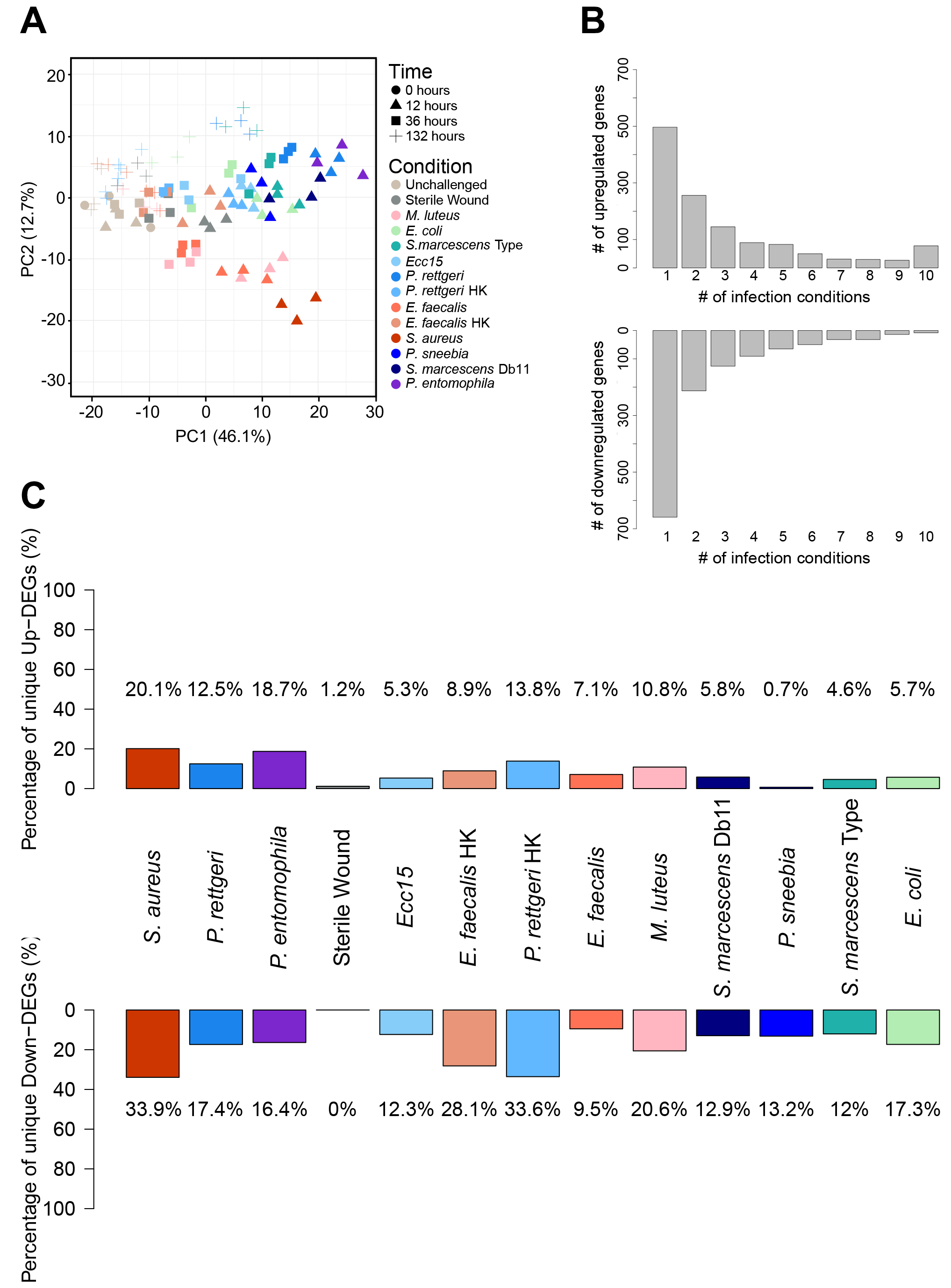

Supplement: S3 Fig — (A) PCA plot showing the first two principal components of the entire dataset. Pink, orange, and red (warm) colors show infections with Gram-positive (Lys-type PGN) bacteria, while green, blue, and purple (cool) colors denote infections with Gram-negative (DAP-type PGN) bacteria. HK indicates stimulation with heat-killed bacteria. (B) Histogram of differentially upregulated (top) or downregulated (bottom) genes by the number of infection conditions in which a given gene was differentially expressed. (C) Percentage of genes that are uniquely upregulated (top) or downregulated (bottom) by each infection. (TIF) [file ppat.1006847.s003.tif]

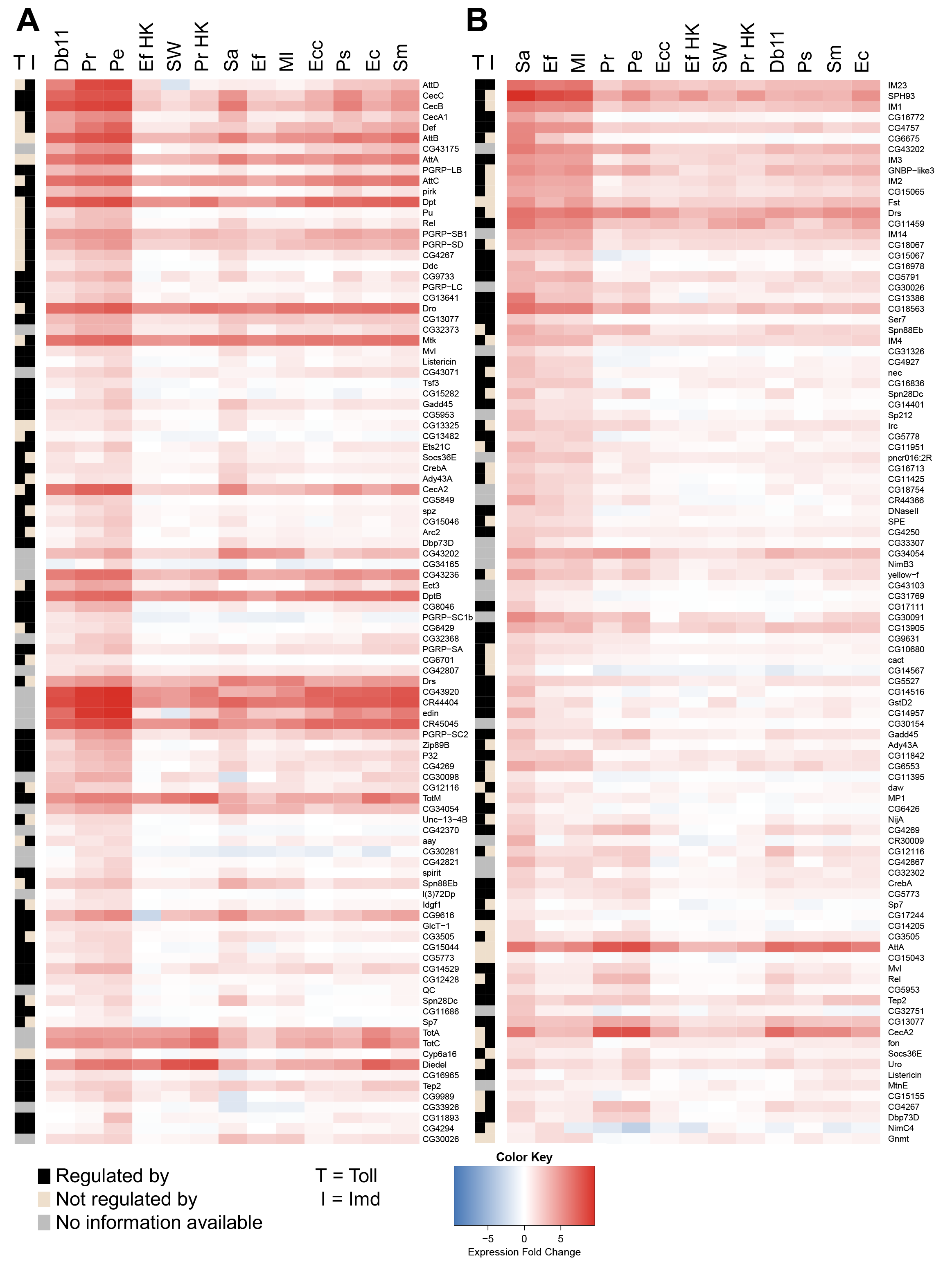

Supplement: S4 Fig — Heatmap (log2 fold change) of top 100 genes that contribute the most to PC1 (A) and of top 100 genes that most contribute to PC2 (B). A gene was deemed to be regulated by the Toll or Imd pathways if the absence of the key genes in each pathway (spz for Toll and Rel for Imd) changed the expression level of said gene by 20% or more compared to the expression level of the gene in wildtype, as previously reported [7]. A color scale on the left side of each heatmap indicates whether each gene is regulated by Toll (T) or Imd (I). In the first column (T), genes regulated by the Toll pathway are marked in black, while genes not regulated by Toll are marked in beige. Similarly, in the second column (I), genes regulated by Imd are marked in black, while genes not regulated by Imd are marked in beige. Genes were marked in gray when no information was available about their regulation by Toll or Imd. (TIF) [file ppat.1006847.s004.tif]

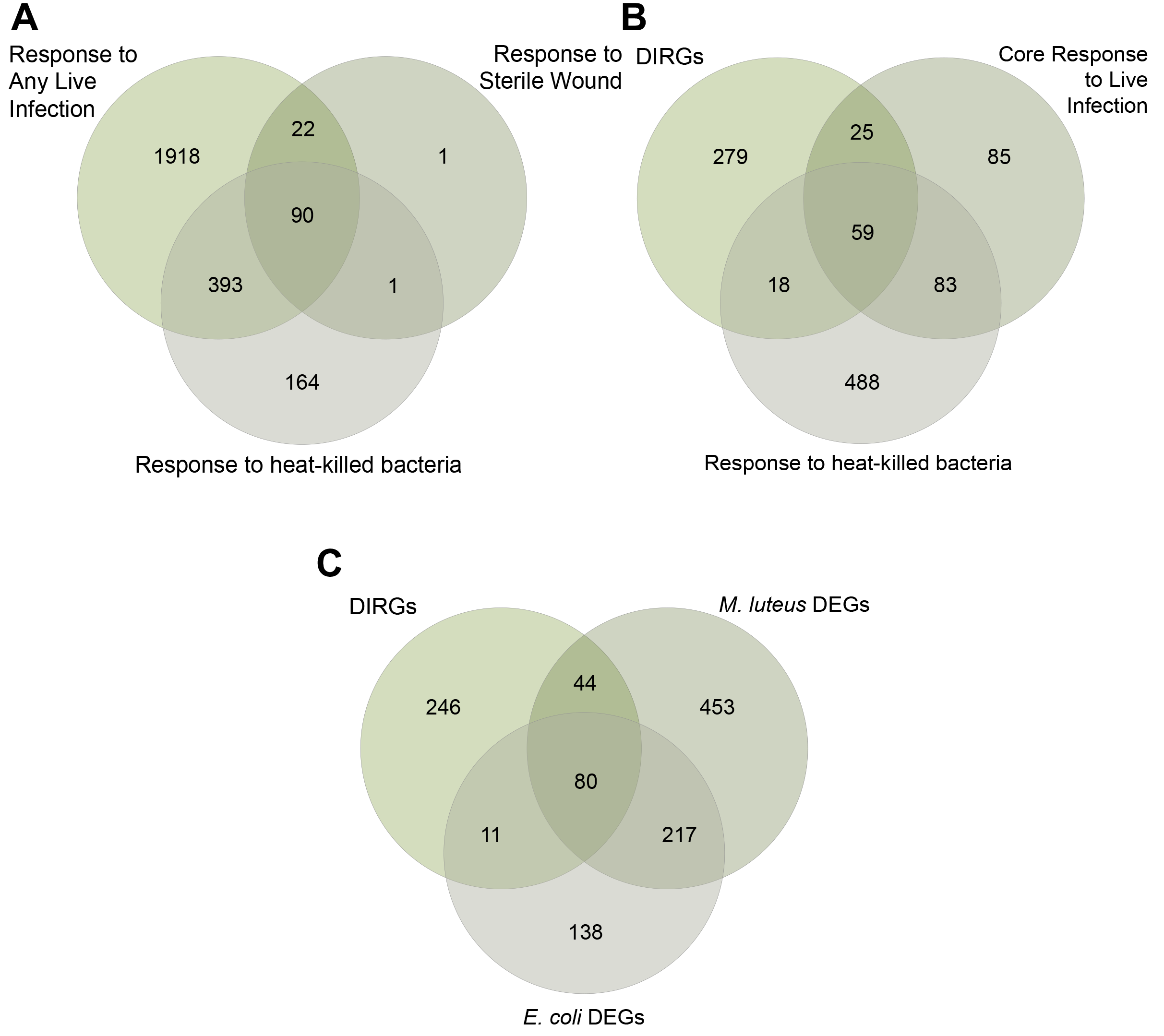

Supplement: S5 Fig — (A) Venn diagram showing the intersection between genes that are differentially regulated in response to at least one live infection, sterile wound, and challenge with heat-killed bacteria. (B) Venn diagram depicting the overlap between the previously described Drosophila Immune-Regulated Genes (DIRGs) [6], core genes differentially regulated in response to live infection, and genes differentially expressed in response to heat-killed bacteria. (C) Venn diagram illustrating the overlay between genes differentially regulated in response to M. luteus infection and, separately, E. coli infection in the present study and the DIRGs, which were previously identified from infection with a mixed cocktail of E. coli and M. luteus [6]. (TIF) [file ppat.1006847.s005.tif]

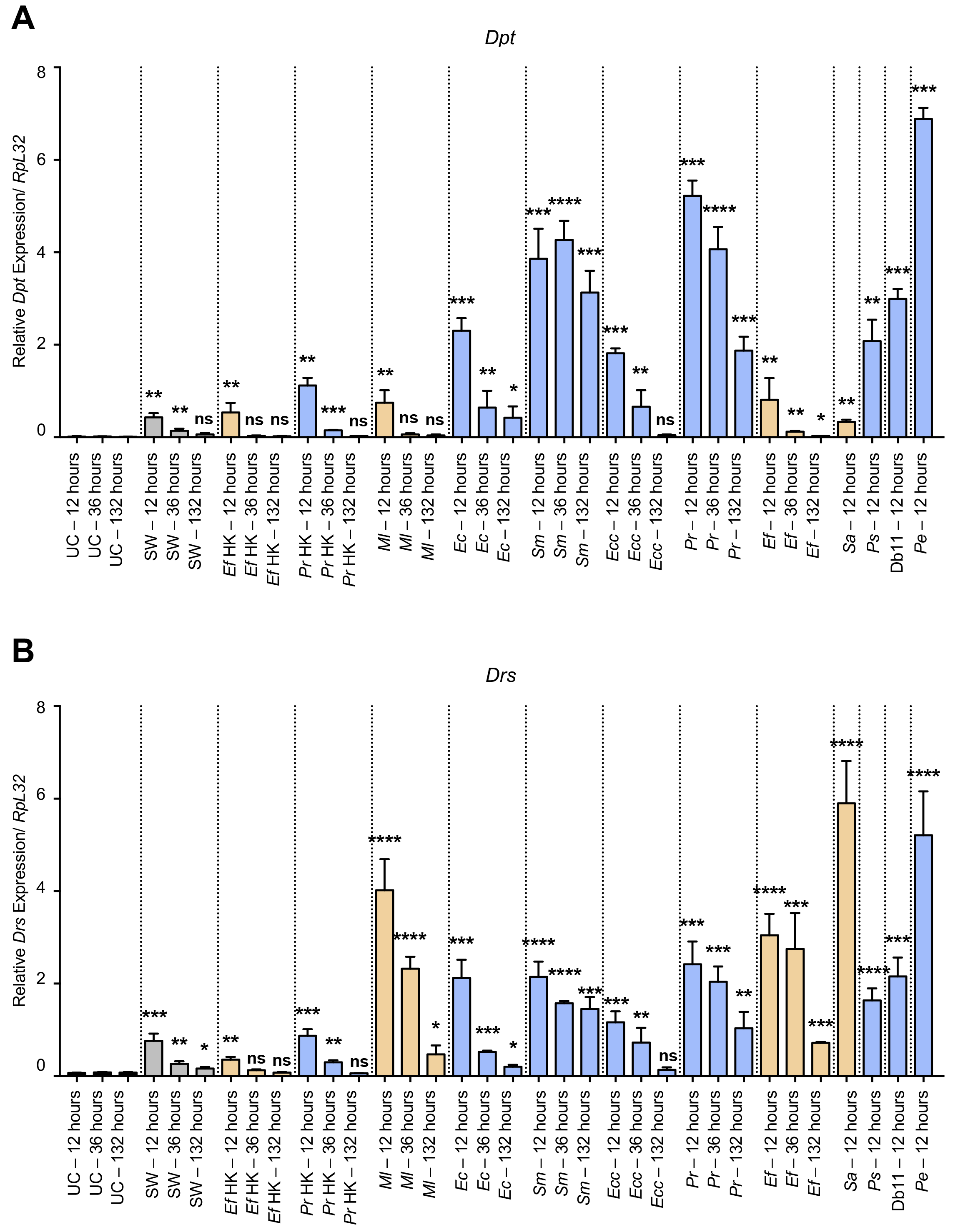

Supplement: S6 Fig — RT-qPCR measuring (A) Diptericin and (B) Drosomycin expression levels in control and infected Canton S flies at 12, 36, and 132 h post-infection. These samples are separate biological replicates, distinct from those used in the RNA-seq experiment. Mean values of three biological repeats are represented ±SE. *p<0.05 **p<0.01 ***p<0.001 ****p<0.0001 in a Student’s t-test. (TIF) [file ppat.1006847.s006.tif]

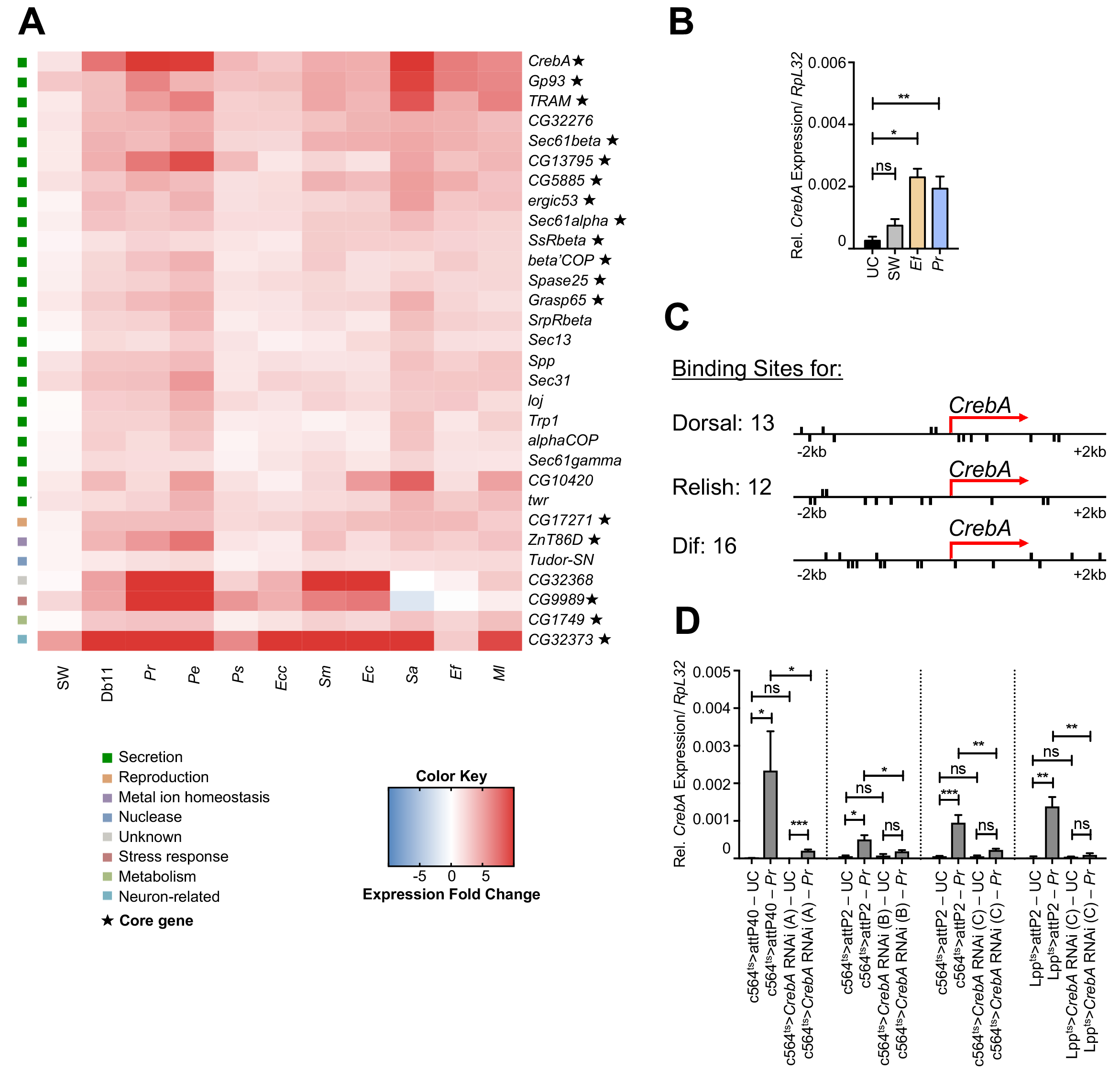

Supplement: S7 Fig — (A) Heatmap showing the expression levels (log2 fold change) of a select group of putative CrebA target genes found to be significantly upregulated by infection. Core genes (marked by a ★) and their functions are highlighted. (B) RT-qPCR validation of CrebA induction levels 12 h after infection with P. rettgeri (Pr) and E. faecalis (Ef) using samples distinct from those used in the RNA-seq. (C) Schematic of predicted Dif, Dorsal, and Relish binding sites on the CrebA promoter region (+/-2kb from the start site). (D) Whole fly RT-qPCR of flies with CrebA knockdown in the fat body following infection with P. rettgeri. CrebA RNAi (A), (B), and (C) denote three distinct RNAi constructs used to target CrebA mRNA. Mean values of three or more repeats are represented ±SE. *p<0.05 **p<0.01 ***p<0.001 in a Student’s t-test. (TIF) [file ppat.1006847.s007.tif]

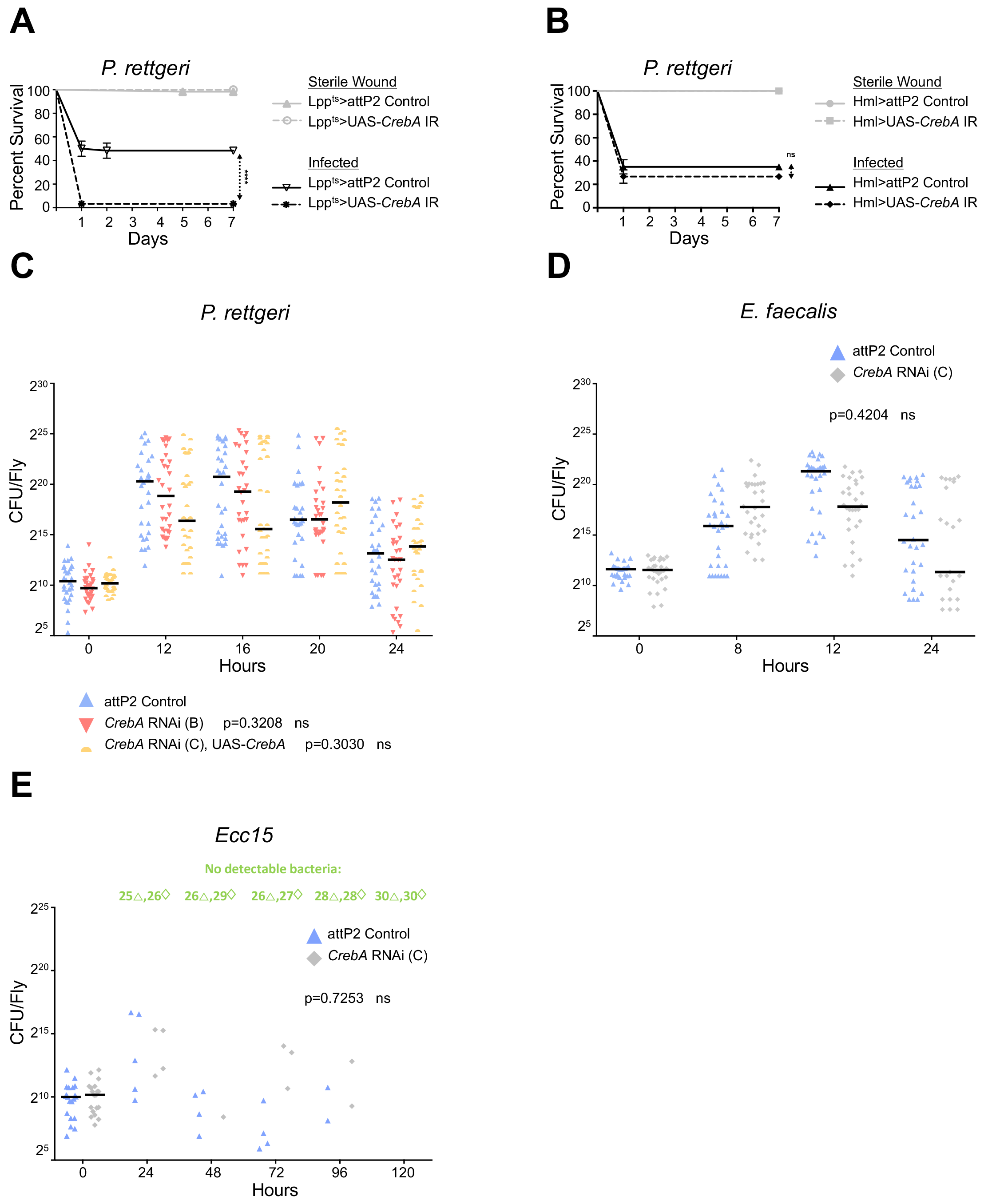

Supplement: S8 Fig — (A) Survival curves over 7 days following P. rettgeri infection of flies whose expression of CrebA is blocked with RNAi specifically in the fat body with a second driver, Lpp-Gal4 (Gal80ts; Lpp-Gal4 > UAS-CrebA-IR). attP2 is the background genotype control, in which CrebA is fully expressed. (B) Survival of unchallenged and infected (P. rettgeri) control flies and flies expressing CrebA RNAi in hemocytes only (Hml-Gal4 driver). The curves represent the average percent survival ±SE of three biological replicates. ****p<0.0001 in a Log-rank test. (C) Bacterial load time course of control flies, flies expressing a separate CrebA RNAi construct (construct B), and flies simultaneously co-expressing a CrebA RNAi and a CrebA overexpression construct in the fat body following infection with P. rettgeri. Bacterial load time course of CrebA knockdown and control flies after infection with (D) E. faecalis and (E) Ecc15. Three repeats are graphed together, with each symbol representing an individual fly’s number of colony forming units (CFU). Horizontal lines represent median values for each condition. A number followed by the symbol Δ (attP2 control flies) or the symbol ♢ (CrebA RNAi flies) indicates the number of flies found to have no bacteria (flies that carry undetectable levels of bacteria or that have cleared the infection) at the specified time point. (TIF) [file ppat.1006847.s008.tif]

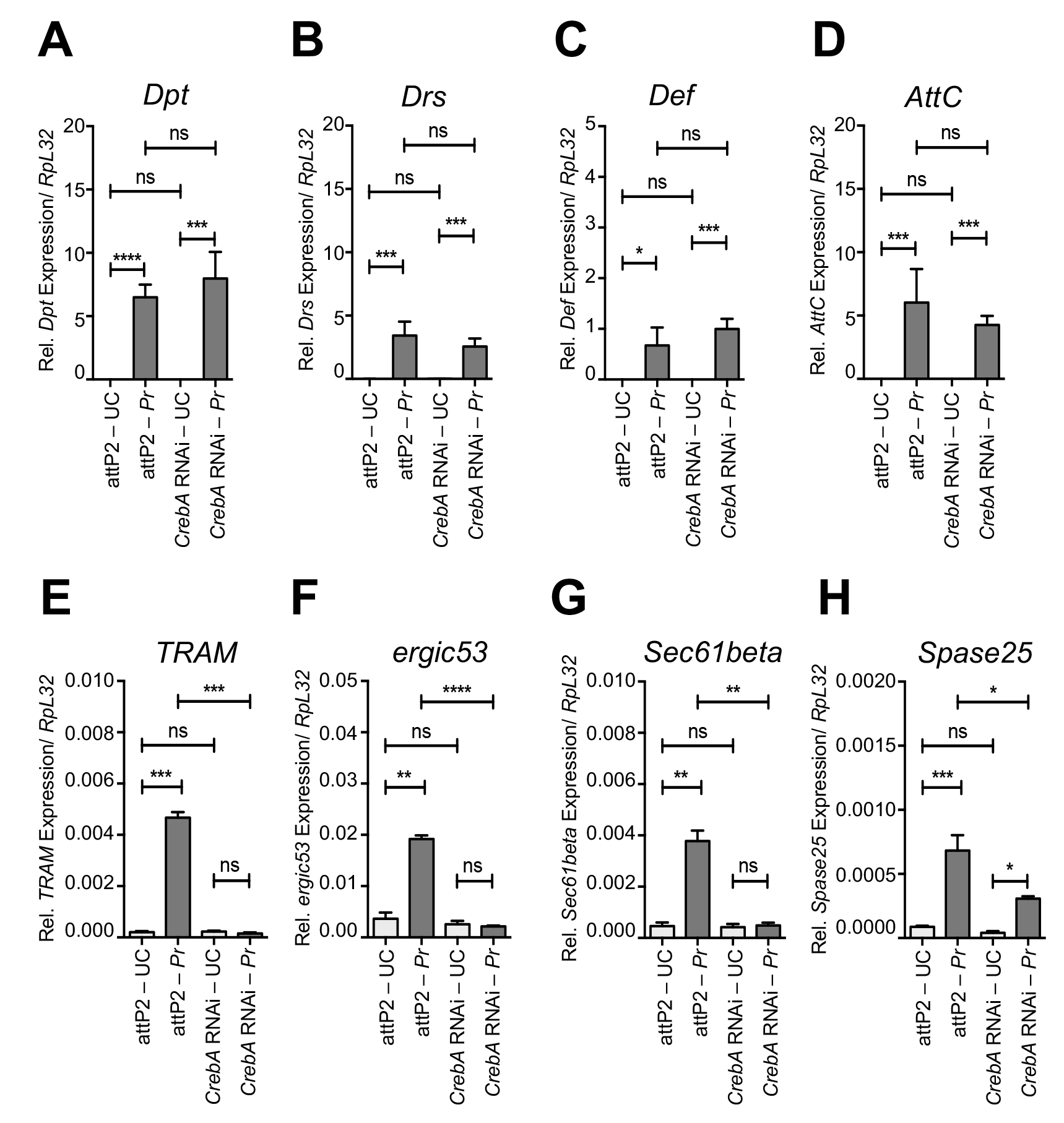

Supplement: S9 Fig — Expression level of predicted CrebA target genes in unchallenged (UC) or infected (Pr) conditions in control (attP2) and CrebA RNAi fat body samples. Assayed genes encoding antimicrobial peptides are (A) Diptericin, (B) Drosomycin, (C) Defensin, and (D) Attacin C. Surveyed genes encoding secretory factors are (E) TRAM, (F) ergic53, (G) Sec61beta, and (H) Spase25. Mean values of three biological replicates are represented ±SE. *p<0.05 **p<0.01 ***p<0.001 ****p<0.0001 in a Student’s t-test. (TIF) [file ppat.1006847.s009.tif]

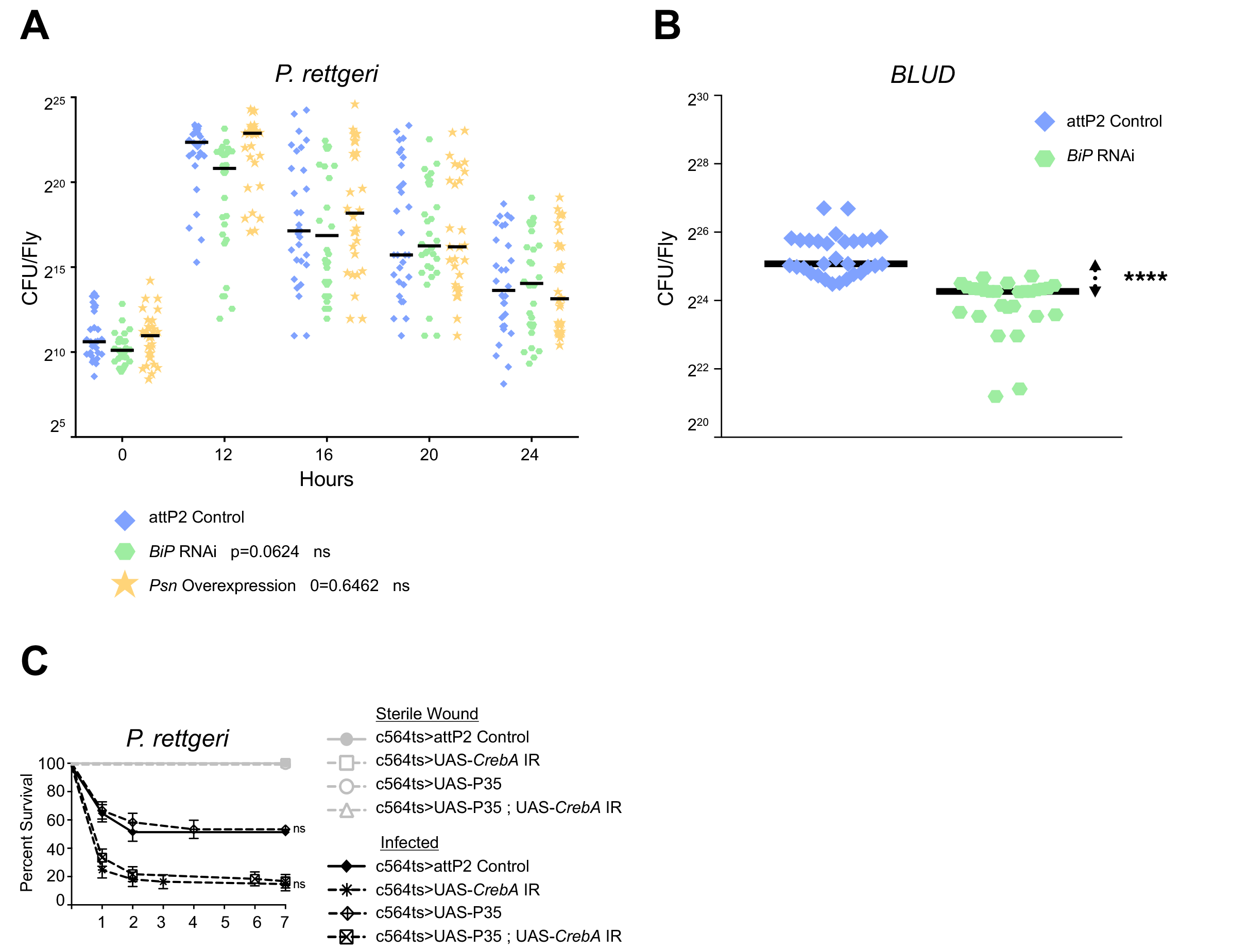

Supplement: S10 Fig — (A) Bacterial load time course of control flies and flies expressing BiP RNAi or Psn overexpression in the fat body following infection with P. rettgeri. (B) Bacterial load upon death (BLUD) following P. rettgeri infection of wildtype controls and flies with BiP expression knocked down by RNAi in the fat body. Three repeats are graphed together, with each symbol representing an individual fly’s number of colony forming units (CFU). Horizontal lines represent median values for each condition. ****p<0.0001 in a Student’s t-test. (C) Survival curves of flies co-expressing CrebA RNAi and the apoptosis inhibitor P35 in fat body cells. The curves represent the average percent survival ±SE of three biological replicates. (TIF) [file ppat.1006847.s010.tif]
